# Selective CO_2_ Adsorption in Ultrahydrophobic Molecular Pyrene Frameworks by Computational Design

**DOI:** 10.1021/jacs.5c06861

**Published:** 2025-06-20

**Authors:** Sam D. Harding, Tao Liu, Linjiang Chen, Siyuan Yang, Isaiah Borne, Thomas Fellowes, Aaron W. Peters, Simon C. Weston, John W. Ward, Andrew I. Cooper

**Affiliations:** † Materials Innovation Factory, Department of Chemistry, 4591 The University of Liverpool, 51 Oxford Street, Liverpool L7 3NY, U.K.; ‡ ExxonMobil Technology and Engineering Company, 22777 Springwoods Village Parkway, Annandale, New Jersey 08801, United States; § Leverhulme Research Centre for Functional Materials Design, The University of Liverpool, 51 Oxford Street, Liverpool L7 3NY, U.K.

## Abstract

The separation of carbon dioxide from industrial flue gas streams using porous materials is often thwarted by humidity. Most porous sorbents adsorb water more effectively than CO_2_. Hence, water can out-compete CO_2_ for adsorption sites, lowering the working CO_2_ sorption capacity and increasing sorbent regeneration costs. Here, two pyrene-based hydrogen bonded organic frameworks (HOFs) are described that can separate CO_2_ under humid conditions. The framework building blocks were chosen in a high-throughput density functional theory screen, followed by crystal structure prediction (CSP) to target a hydrophobic two-dimensionally porous framework. Gas sorption experiments showed selective adsorption of CO_2_ and exceptionally low water adsorption in these HOFs. Dynamic column breakthrough measurements using mixed gas environments showed that the CO_2_ working capacity was totally unaffected by water under simulated flue gas conditions up to 75% relative humidity. One of the CO_2_-selective HOFs, diMeTBAP-α, was shown by CSP to be the most thermodynamically stable structure on the crystal energy landscape. This stability prediction was reflected by experiments, where an isostructural, scalable analogue of diMeTBAP-α, MeTBAP-α, retained its porosity and crystallinity after boiling in aqueous acids, which is important for carbon capture from acidic, humid flue gas.

## Graphical abstract

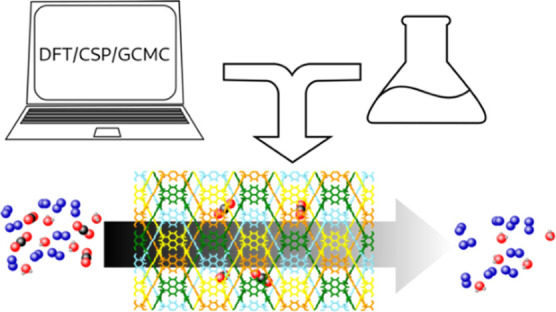

## 1 Introduction

Anthropogenic climate change is a major challenge for society.^1^ To tackle it, we need alternative energy sources, such as renewables, nuclear, and hydrogen power, but these have their own technical problems that must first be overcome before they can satisfy global energy demand.^2–4^ In the meantime, interim technologies such as carbon capture and storage (CCS) are needed to reduce CO_2_ emissions.^5^

There are several relevant methods for CCS, including oxyfuel combustion, direct air capture (DAC), bioenergy with CCS (BECCS) and point capture. Due to the higher concentration of CO_2_ and the ease of retrofitting existing equipment, the main large-scale industry focus is point capture from flue gas.^6^ At present, this is mostly achieved by amine scrubbing, but this has drawbacks including high regeneration costs, emissions related to degradation products, and corrosion of equipment.^7^ We therefore need efficient, less energy-intensive ways of capturing CO_2_ from flue gas. One possible solution is physisorption in microporous solids, which can have internal surface areas as high as 7000 m^2^ g^–1^ and tunable physicochemical properties.^6^ Most microporous materials are extended solids linked by coordination or covalent bonds, such as zeolites,^8,9^ metal–organic frameworks (MOFs),^10–12^ covalent organic frameworks,^13,14^ hyper-cross-linked polymers,^15^ and conjugated microporous polymers.^16,17^ Porous solids can also be constructed from molecular building blocks using weaker, more labile interactions such as in hydrogen bonded organic frameworks (HOFs),^18,19^ supramolecular organic frameworks,^20,21^ porous organic cages,^22,23^ and nonmetal organic frameworks.^24^

However, while a huge range of microporous materials now exists, there are challenging requirements for CCS, such as structural stability and maintaining CO_2_ capacity and selectivity in an acidic, humid flue gas environment.^25^ It is also important to manage the CO_2_ binding energy to adsorb the gas while also providing economically feasible regeneration energies.^26^

For point capture CCS, the typical composition of the flue gas is around 3–15% CO_2_, 3–12% O_2_, and 8–10% H_2_O, the remainder being made up of N_2_ with trace gaseous acids and particulates.^6^ The presence of water is particularly challenging: it is much more polar than CO_2_ and therefore tends to adsorb more strongly in porous solids.^27^ To be viable for CCS, porous sorbents must be as selective toward CO_2_ over water as possible.

One material that has been explored extensively in this regard is the zinc-based MOF, CALF-20.^28^ CALF-20 shows excellent CO_2_ capacity and selectivity for CO_2_ over water at low humidities and the material has been produced on a pilot plant scale.^29^ While CALF-20 is one of the leading materials in this field, there are still challenges, such as its poor performance at lower CO_2_ partial pressures (with N_2_ present) at >40% relative humidity (RH).^30^ Hence, there has been much interest in developing new materials that perform under the higher relative humidities (>70% RH) that are typically observed in flue gas.^6^ In this respect, aluminum formate (ALF) frameworks and their iron containing derivatives (Fe-ALF) have recently shown great potential, and they promise to be scalable.^11,12^

Ultimately, the selectivity between CO_2_ and water stems from atomistic scale effects, hence computational chemistry is a powerful way to design porous solids.^31–33^ HOFs are interesting candidates because they are composed of discrete organic molecules that form rigid framework structures upon crystallization, and importantly they also lack potentially polar metal centers that could serve as binding sites for water.^34^ As a result, we can gain some useful chemical information from the molecular building blocks considered in isolation. Moreover, it has been shown that both the crystal structure and gas sorption properties for HOFs can be predicted using a priori crystal structure prediction (CSP) methods.^35–37^ Thus, computational design has been used to access HOFs with high surface areas,^35^ photocatalytic activity,^36^ and high gas storage capacities.^37^

Here, we used bottom-up computational screening of molecular fragments (Figure 1), followed by crystal structure prediction (Figure 2) to predict the framework structure. Based on these predictions, we then used experimental synthesis to access two HOFs for selective CO_2_ capture under high humidity environments. First, density functional theory (DFT) methods^38^ were used to calculate CO_2_ and H_2_O binding energies to identify the best molecular building blocks, which led us to consider pyrene-based HOFs. CSP calculations^35–37,39^ were then used to predict the crystal structures and the porous properties for the candidate HOFs.^40^ Finally, Grand Canonical Monte Carlo (GCMC)^41^ methods were used to model CO_2_ capacities for the predicted porous HOF phases and to construct energy-structure–function (ESF)^37^ maps. The most promising framework identified in silico (diMeTBAP-α) was then prepared experimentally, along with a more synthetically scalable structural analogue (MeTBAP-α). The CO_2_ and H_2_O adsorption properties of these HOFs were then tested. In single component sorption experiments, diMeTBAP-α and MeTBAP-α showed CO_2_ uptakes of 1.95 and 1.64 mmol g^–1^ at 1 bar and 298 K, respectively. Further, due to the highly aromatic and hydrophobic internal surfaces of the pore, these materials display water uptakes that are remarkably low compared to other porous solids, with uptakes of 1.30 and 1.85 mmol g^–1^ (298 K, 32 mbar), respectively. Under more practically relevant mixed gas dynamic conditions, these acid-stable HOFs showed absolutely no reduction in CO_2_ capacity in the presence of water over multiple cycles.

**Figure 1:**
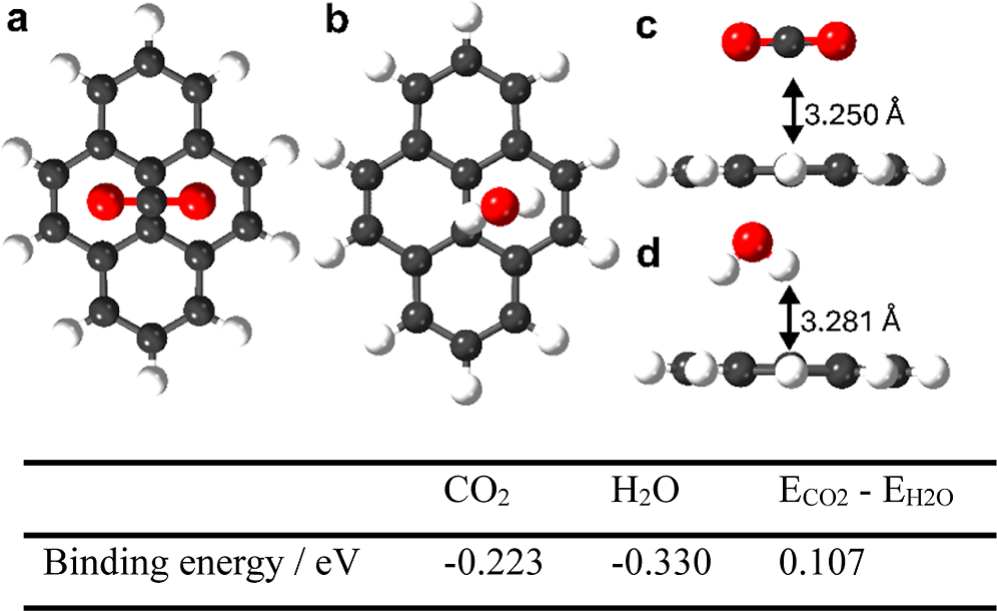
Predicted lowest energy intermolecular interactions of an isolated pyrene molecule in the gas phase with single CO_2_ and H_2_O molecules. The table summarizes the calculated binding energies, and the difference between the CO_2_ and H_2_O binding energy, *E*_CO_2__-*E*_H_2_O_. The energetic preference for water binding in the gas phase (0.107 eV) was smaller than calculated for most of the 27,446 molecular fragments that were screened (rank 6th).^42^

**Figure 2:**
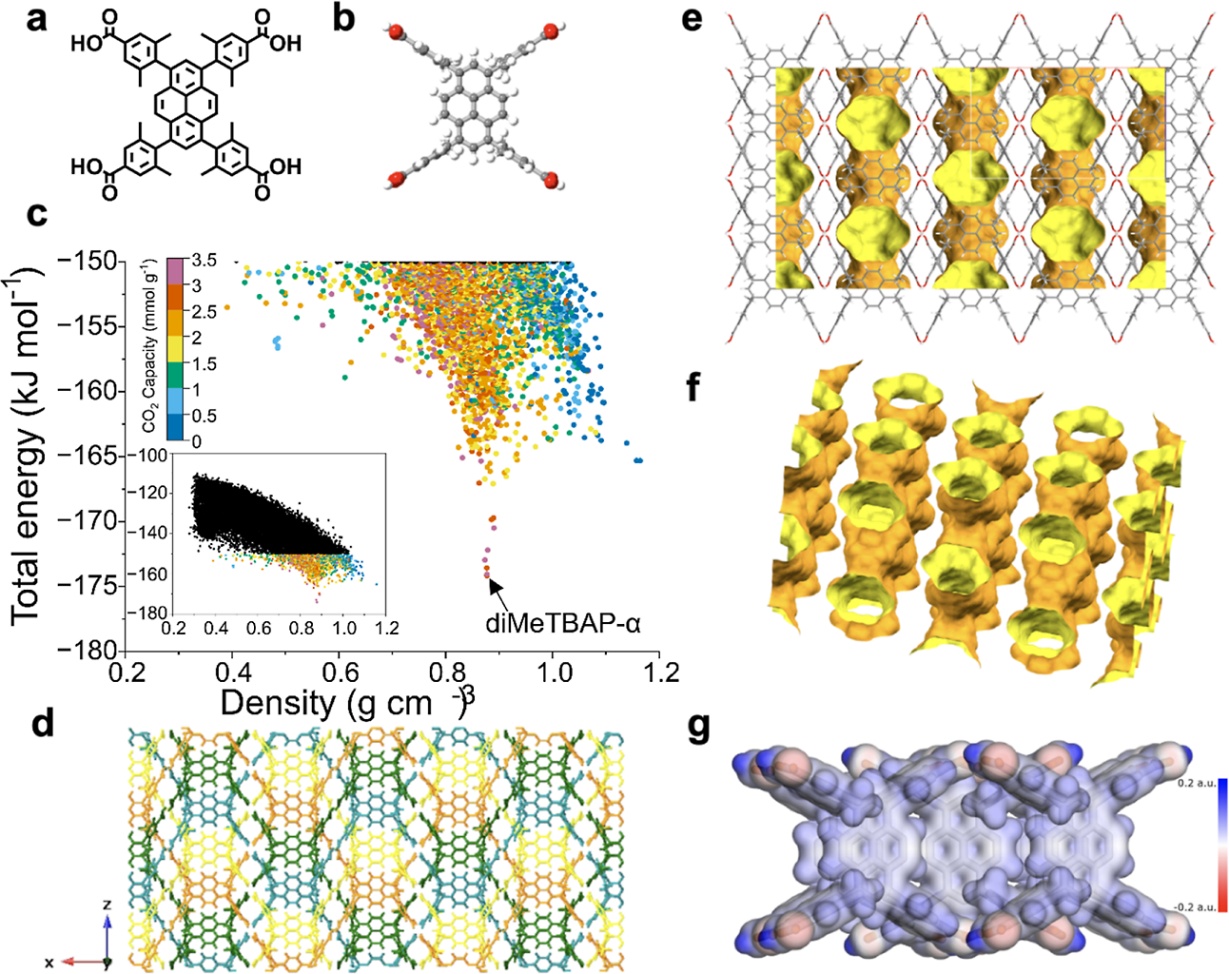
Energy-structure–function maps predict a thermodynamically stable porous diMeTBAP crystal with hydrophobic 2-D pore channels and good CO_2_ sorption capacity. (a), Molecular structure and (b), calculated stable conformer of diMeTBAP. (c), ESF map displaying the computed densities, lattice energies, and dry CO_2_ capacities (298 K, 1 bar) for diMeTBAP; CO_2_ capacities were only calculated for structures in the CSP data set (inset) with lattice energies below 150 kJ mol^–1^. (d), 3-D representation of the lowest energy crystal structure found, diMeTBAP-α, showing the arrangement of the interpenetrated diMeTBAP layers (see also Figure S14). (e), diMeTBAP-α has 2-D pore layers, as shown here using a 1.2 Å probe size (yellow = interior of pores, orange = exterior). The polar carboxylic acids (red atoms represent oxygens) are buried between these 2-D pore layers. (f), Isometric view of the 2-D pore layers; diMeTBAP molecules omitted for clarity. (g), Calculated electron density isosurface showing low charge density in pores of an isomorphous analogue, MeTBAP-α (Section S1.5 for details). The highly charged carboxylic acid groups are shieled by the methyl groups, so the overall pore surface is hydrophobic (scale ranges from −0.2 au to 0.2 au).

## 2 Results and Discussion

### 2.1 Computational Screening of Molecular Building Blocks

Pyrene emerged as a possible CO_2_-selective building block from a high-throughput fragment screening workflow based on DFT methods.^42^ Briefly, a total of 27,446 molecular fragments were screened to determine CO_2_ and H_2_O binding energies for each isolated fragment in the gas phase. This was done by docking single sorbate molecules with the molecular fragments and then optimizing the structures using DFT with B97D3 functional^38,43^ and def2-svp basis set^44^ implemented in Gaussian16.^45^ Pyrene derivatives were one promising set of molecules identified in this bottom-up computational screening analysis.^42^ As a result of their relatively large π-systems, pyrene was predicted to physisorb CO_2_ with relatively good selectivity against water (Figure 1), at least for isolated pyrene molecules in the gas phase. Pyrene was still predicted to bind water more strongly than CO_2_ in the gas phase (*E*_CO_2__-*E*_H_2_O_ = 0.107 eV), but the energy difference was lower than the vast majority of the other 27,446 molecular fragments screened, most of which favored water binding strongly.^42^ It was therefore reasoned that porous materials with multiple pyrene systems might be selective for the sorption of CO_2_ over water. This computationally led hypothesis is supported by the earlier work of Boyd et al., who demonstrated that parallel pyrene systems in MOFs with *a* spacing ca. 7–8 Å acted as effective CO_2_ binding sites.^46^ Effectively, pyrene units re-emerged here in our unbiased, bottom-up computational screen of 27,446 molecular fragments.^42^

We next set about designing metal-free pyrene HOFs with a geometric arrangement of pyrene units that would express this predicted gas-phase CO_2_/H_2_O selectivity. One known pyrene HOF is the α-polymorph of 1,3,6,8-tetrakis­(4-benzoic acid)-pyrene (TBAP), first reported by some of us.^36^ This HOF has one-dimensional (1-D) pore channels and surface areas of up to 1500 m^2^ g^–1^. However, because of the π-stacking exhibited between the pyrene cores in crystalline TBAP-α, the faces of the pyrene rings are not exposed to the internal pore surface of the HOF (Figure S15), thus preventing the CO_2_ binding mode predicted in Figure 1. Moreover, the 1-D pores in TBAP-α are lined with polar carboxylic acid dimers, and hence this HOF exhibits high water uptakes (>14 mmol g^–1^ at 32 mbar/298 K). This is analogous to the problem with many MOFs where the organic linker may be nonpolar, but the metal binding units, such as carboxylic acids or amines, are not.

Previous work by Nandi et al.,^19^ Yang et al.,^47^ and Wang et al.^48^ have shown that alternative HOF topologies can be accessed by disrupting π-stacking. It was therefore reasoned that adding one or more methyl groups ortho to the phenyl-pyrene bond of TBAP might generate an alternative three-dimensional (3-D) structure, thus exposing the pyrene π-systems within the pores for CO_2_ binding. In principle, the addition of methyl groups also has the potential to increase hydrophobicity, as demonstrated by Wang et al., who introduced methyl groups into CALF-20.^49^

With this in mind, we considered a methylated derivative of TBAP, 1,3,6,8-tetrakis­(2,6-dimethyl-4-benzoic acid)-pyrene (diMeTBAP) (Figure 2a). The diMeTBAP molecule was predicted to have one stable conformer (Figure 2b) where the steric bulk of the methyl groups cause the four benzoic acid arms to twist by 90° out of the plane of the pyrene ring. It seemed clear that this 90° twist had the potential to break up π-stacking: beyond this, it was impossible for us to anticipate the low-energy crystal packing of diMeTBAP, nor whether it would form a stable porous crystal that did not expose the polar carboxylic acid groups, as observed in TBAP-α (Figure S14), thus binding water more effectively than CO_2_.

To assess the potential of diMeTBAP for CCS, its crystal packing was explored by de novo CSP with the DREIDING force field. This generated 35,324 crystal structures along with their associated lattice energies (Figure 2c). The energy-density landscape for diMeTBAP was dominated by a pronounced ‘spike’ at a density of around 0.88 g cm^–3^. At the tip of this spike was the global minimum predicted structure, diMeTBAP-α, which was porous. This material was predicted to have two planes of interpenetrated AB-stacked layers (Figure 2d) featuring a 2D network of pores with a width of ∼4.5 Å (Figure 2e,f). Other HOFs that showed porous spikes on their CSP landscapes have yielded porous structures by experiment,^36,37^ but those porous phases were predicted to be metastable with respect to denser, nonporous crystal packings. The presence of a porous global minimum structure, diMeTBAP-α, is especially promising because it suggests that diMeTBAP-α might be stable for practical applications.^24^ That is, there are no denser, more stable crystal structures available for this material to collapse to, which is a well-documented problem with metastable HOFs and MOFs.^50–53^

In addition to being porous, the predicted crystal packing of diMeTBAP-α suggested its potential for selective CO_2_ capture in the presence of water. In this low-energy predicted crystal packing, the polar carboxylic acids are buried between the 2-D pore layers (Figure 2e,f). The resulting pores were therefore predicted to be hydrophobic, being dominated by exposed pyrene units with low calculated electrostatic potentials (Figure 2g). By contrast, TBAP-α has a crystal packing where the pyrene faces are pi-stacked and hidden, and where the hydrogen-bonded carboxylic acid dimers line the 1-D pore structures. This means that TBAP-α is a relatively hydrophilic material, which is useful for other applications, such as photochemical water splitting,^37^ but not for selective CCS under humid conditions.

To assess viability for CO_2_ adsorption, CO_2_ capacities at 298 K were simulated up to 1 bar using GCMC (Section S1.3), with the DREIDING force field for only the 3384 structures with predicted lattice energies below 150 kJ mol^–1^ for computational affordability. The CO_2_ uptakes at 1 bar were then used to generate the energy-structure–function (ESF) map^37^ shown in Figure 2c. The predicted CO_2_ capacity for perfectly crystalline, defect-free diMeTBAP-α was 3.3 mmol g^–1^ at 298 K and 1 bar. Denser, nonporous structures with no CO_2_ uptake were also found on the ESF map (Figure 2c), but these were predicted to have much higher lattice energies and, hence, deemed unlikely to be formed. As such, de novo computational predictions suggest that diMeTBAP might express good CO_2_ uptakes, low water sorption, and high physicochemical stability in the solid crystalline state.

### 2.2 Synthesis of Predicted Porous Pyrene HOFs

To test our computational predictions, we synthesized the monomer diMeTBAP and its corresponding HOF. The two methyl groups ortho to the pyrene-aryl bond create a sterically hindered environment, and this resulted in poor yields for both Suzuki coupling and Miyaura borylation of the starting bromide (17% and 32%, respectively, leading to an overall yield of 5%). These low yields clearly pose a barrier to using this material at scale, and while alternative catalysts such as the PEPPSI-IPr catalyst have proven effective for the Suzuki coupling of other hindered aromatics, we were unable to improve yields in this manner.^54^ Therefore, to reduce the effects of this steric hindrance,^55^ we also investigated 1,3,6,8-tetrakis­(2-methyl-4-benzoic acid)-pyrene (MeTBAP) as an alternative, less hindered analogue (Figure 3). MeTBAP could be synthesized in three steps in much higher overall yield (63% vs 5%) at gram scales via Suzuki coupling, making it easier to scale this material for gas sorption experiments, although it remains more costly than certain framework materials, such as ALF.^11^

**Figure 3:**
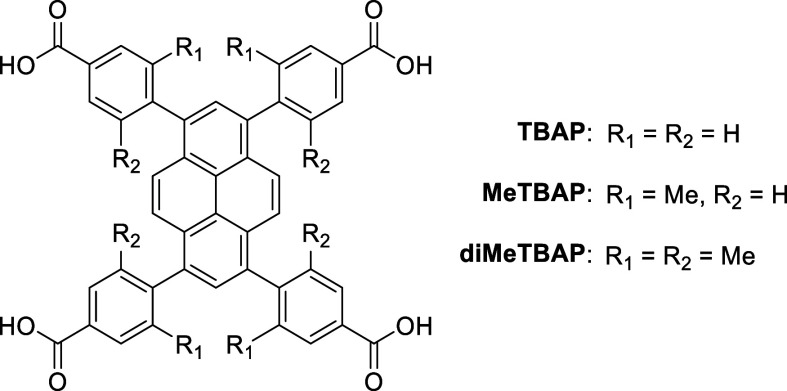
Chemical structures of TBAP, MeTBAP and diMeTBAP. The less sterically hindered targets, TBAP and MeTBAP, give much higher reaction yields.

Analysis of MeTBAP by ^1^H NMR spectroscopy revealed the presence of conformational isomers (Section S4.1) arising from the asymmetry that is introduced by the single methyl group. Variable temperature NMR (VT-NMR) showed coalescence of peaks at 120 °C (Figures S8–S10). The presence of multiple potential isomers made MeTBAP more difficult to explore by CSP, so we did not attempt this; rather, we proceeded on the assumption that MeTBAP might crystallize isostructurally to diMeTBAP-α, prompted by the observation that the carboxylic acids in diMeTBAP, and not the methyl groups, appear to determine its predicted low-energy interpenetrated crystal packing (Figure 2d,e).

Both MeTBAP and diMeTBAP were crystallized by diffusion of chloroform into a saturated dimethylformamide (DMF) solution. The predicted global minimum structure, diMeTBAP-α, was obtained as a polycrystalline powder with a PXRD pattern that agreed with the predicted structure (Figure 4a). No crystals of sufficient size for single crystal XRD (scXRD) could be obtained, thus illustrating the power of CSP for the structure elucidation of new materials. By contrast, MeTBAP-α was obtained both as a polycrystalline powder and as single crystals, allowing detailed structural elucidation. MeTBAP-α was found to be structurally isomorphous with the global minimum CSP-derived structure, diMeTBAP-α (Figure S16). These scXRD data also showed the presence of different isomers of MeTBAP in crystalline MeTBAP-α, leading to populations of syn and anti conformations of the methyl groups, in keeping with the ^1^H NMR data, above. As predicted, these two pyrene derivatives, diMeTBAP and MeTBAP, both yielded isomorphous HOFs by experiment that have 2-D hydrophobic pore channels.

**Figure 4:**
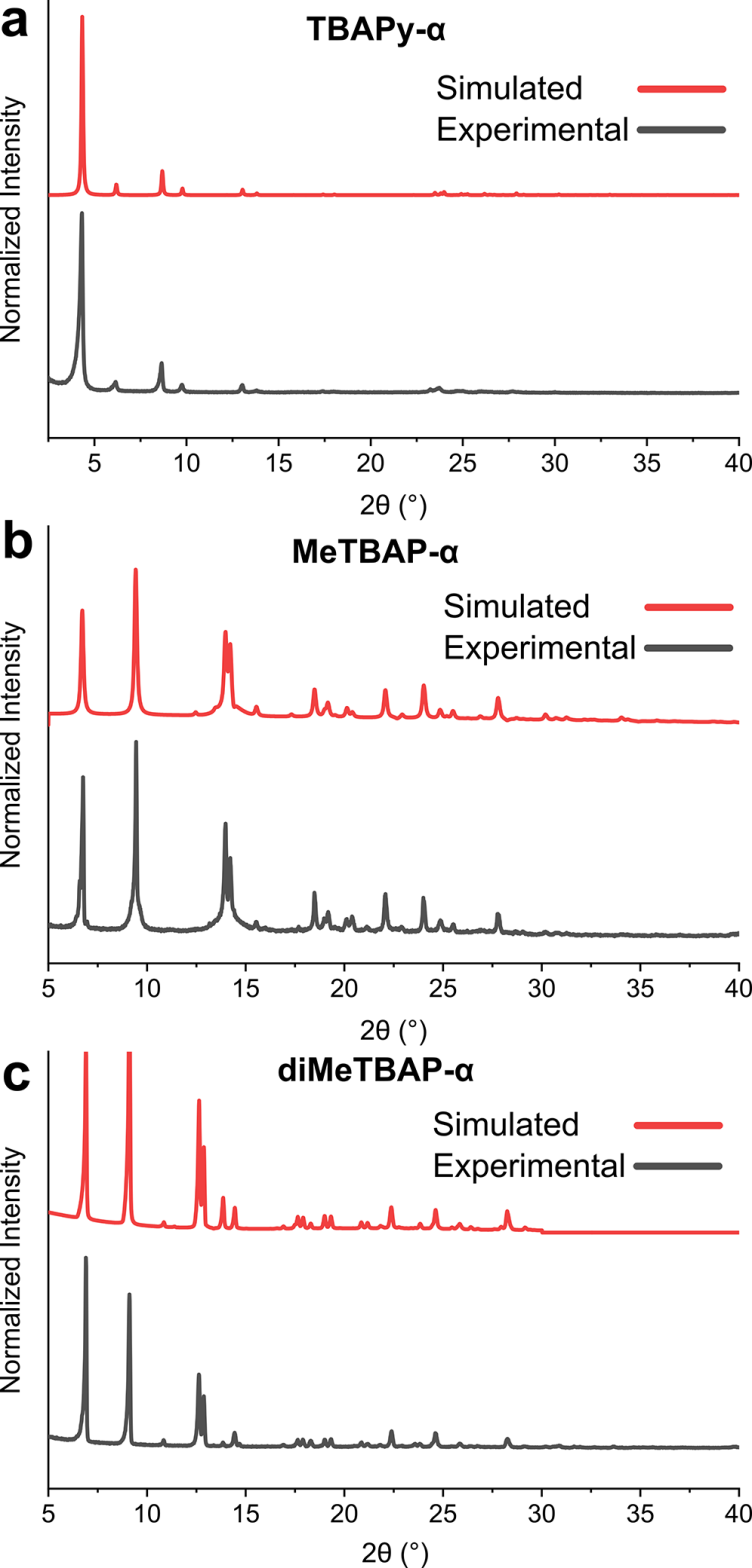
X-ray diffraction confirms formation of the predicted porous HOF phases. (a), Experimental PXRD patterns (black) and pattern simulated from global minimum CSP structure, diMeTBAPy-α (red). (b), Experimental PXRD pattern (black) and PXRD pattern simulated from experimental single crystal data (red) for a DMF solvate of MeTBAP-α. (c), Experimental PXRD pattern (black) and pattern simulated from experimental single crystal data (red) for TBAP-α.^36^

### 2.3 Gas Sorption Properties of TBAP, diMeTBAP and MeTBAP

Having obtained the predicted low-energy porous HOF (diMeTBAP-α) and its isomorphous analogue (MeTBAP-α), we next explored their gas sorption properties (Figure 5). Nitrogen isotherms measured at 77 K (Figure 5a) gave Brunauer–Emmett–Teller (BET) surface areas of 1256 m^2^ g^–1^, 856 m^2^ g^–1^ and 1104 m^2^ g^–1^ for TBAP-α, MeTBAP-α, and diMeTBAP-α, respectively. All three materials showed high gas uptakes and type I sorption isotherms consistent with microporous materials. The experimental pore size distributions showed good agreement with those calculated from structures obtained by CSP (Figure 5b). The dry CO_2_ isotherms at 195 K (Figure S21) gave saturated CO_2_ capacities of 11.52, 8.23, and 10.19 mmol g^–1^ for TBAP-α, MeTBAP-α, and diMeTBAP-α, respectively. Further dry CO_2_ isotherms were also measured at ambient temperatures (273 and 298 K; Figure 5c), and at temperatures closer to those that might be encountered in flue gas environments (313 and 333 K; Figures S25–S26), and isosteric heats of adsorption for CO_2_ were calculated from these isotherms (Figure S28). While the experimental CO_2_ capacity is lower than predicted by GCMC (Figure S27), this is to be expected as the material used is unlikely to be perfectly crystalline.

**Figure 5:**
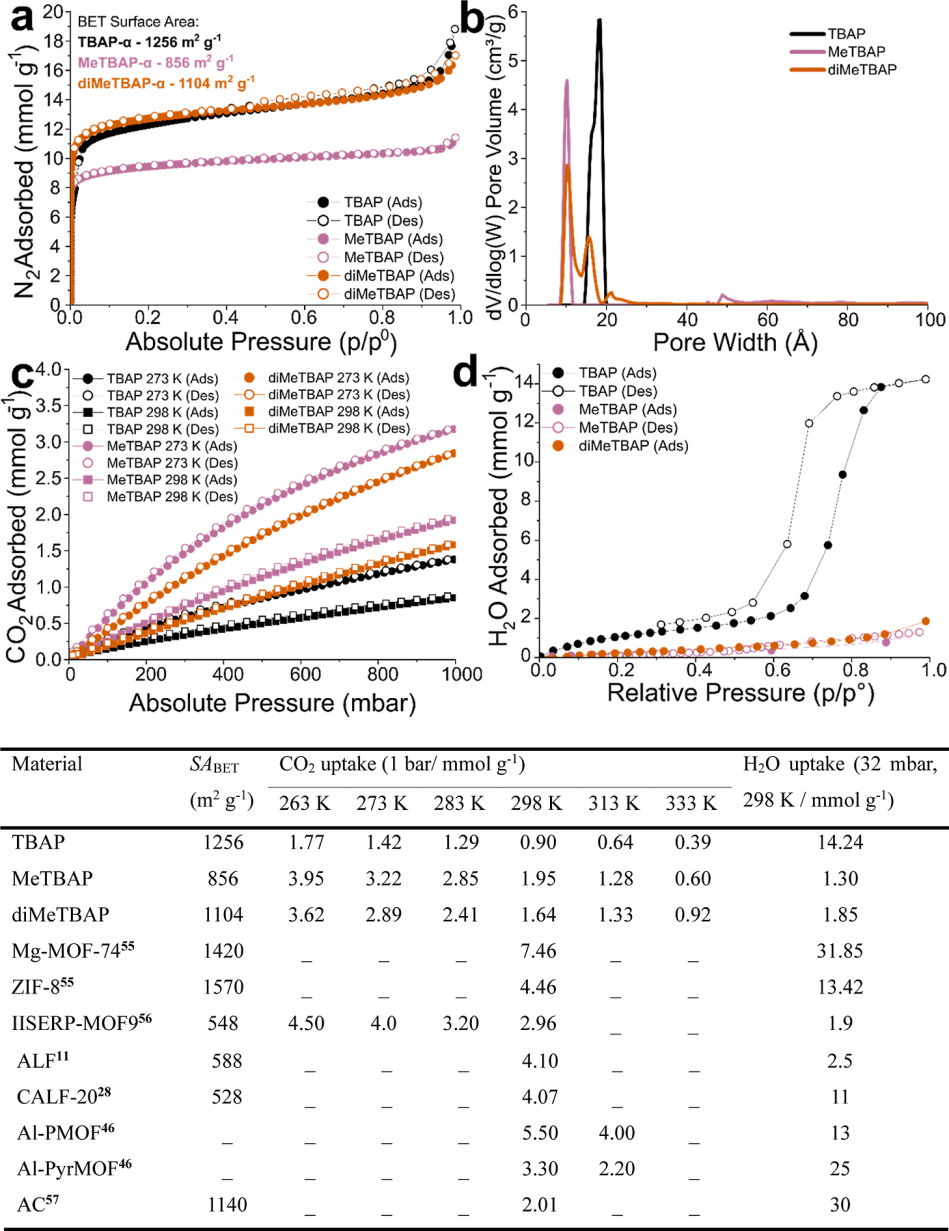
Gas sorption measurements and water uptakes for TBAP-α, diMeTBAP-α and MeTBAP-α. Adsorption and desorption isotherms of TBAP, MeTBAP-α and diMeTBAP-α, including (a), N_2_ at 77 K, (b), DFT pore size distributions calculated from N_2_ isotherms recorded at 77 K, (c), dry CO_2_ uptake at 273 and 298 K, and (d), H_2_O at 298 K. The table summarizes the key sorption metrics for these three pyrene HOFs and for selected benchmark materials (AC = activated carbon); see Table S5 for a fuller list. SA_BET_ = Brunauer–Emmett–Teller surface area.^57^

A low isosteric heat of adsorption was measured for CO_2_ in MeTBAP-α (23 kJ mol^–1^); this is considerably lower than reported for the stable MOF, ALF (47.9 kJ mol^–1^).^11^ While ALF promises to be cheaper, more synthetically scalable, and has a higher native CO_2_ capacity, this shows that ultrahydrophobic aromatic HOFs might have potential to reduce regeneration energies in CCS.

MeTBAP-α and diMeTBAP-α showed dry CO_2_ uptakes that are relatively high for HOF materials^18,19,48,58^ in the temperature range 263–333 K, while TBAP-α showed much lower CO_2_ uptakes (Figure 5, Section S5). Only a few examples of HOFs have been reported to show higher CO_2_ capacities at these temperatures.^18,19,48,58^

To explore the CO_2_/N_2_ selectivity of the material, a nitrogen isotherm at 298 K was recorded for MeTBAP-α, the material adsorbs 0.25 mmol g^–1^ of nitrogen at 298 K and 1000 mbar (Figure S20). The use of ideal adsorbed solution theory to calculate the selectivity of CO_2_ gives a selectivity of 11:1. This selectivity is relatively low compared to many best-in-class materials, a feature that can be attributed to the low isosteric heat of adsorption of the material.

For CCS, performance under humid conditions is more relevant than dry CO_2_ uptakes. Water isotherms were measured at 298 K for TBAP, MeTBAP-α and diMeTBAP-α. The water isotherm measured for TBAP-α was in good agreement with previous reports.^36^ In keeping with the observed crystal structures, TBAP adsorbs much more water (14.24 mmol g^–1^) than its methylated analogues, diMeTBAP and MeTBAP (Figure 5d). Indeed, the water uptakes for MeTBAP-α (1.30 mmol g^–1^) and diMeTBAP-α (1.85 mmol g^–1^) are lower than for most microporous materials reported to date, including relatively hydrophobic materials such as activated carbon (Table, Figure 5 and Table 2). A comparison of CO_2_ uptakes (298 K, 1 bar) and water uptakes (298 K, 32 mbar) for these HOFs and a range of other reported materials is given in Figure S35 and Table S5. MeTBAP-α is one of very few materials reported to absorb more CO_2_ than water under these conditions (Figure S35), at least as assayed from separate CO_2_ and H_2_O isotherms. This suggests potential for high CO_2_ selectivity and low regeneration energies.

### 2.4 Stability of MeTBAP-α under Acidic Conditions

Flue gas contains acidic impurities such as SO_2_ and NO_*x*_, so it is vital that sorbents for CCS are stable under acidic conditions. To probe this, samples of MeTBAP-α were suspended in aqueous 1 M HCl, H_2_SO_4_ or HNO_3_ and left either at room temperature for 3 days or heated to 100 °C overnight. PXRD analyses and gas sorption measurements suggested little to no loss of crystallinity or porosity, demonstrating high stability under acidic environments (Figure S12 and S19). This high stability agrees with the CSP data (Figure 2c), which suggest that there are no more stable crystal packings for MeTBAP-α. We note that these CSP calculations assume both crystallinity and a constant chemical composition. For example, these crystal stability calculations do not consider the possible formation of amorphous phases, chemical decomposition, nor the potential formation of hydrates.

### 2.5 Dynamic CO_2_ Separations Using MeTBAP-α under Humid Conditions

Next, a series of dynamic column breakthrough (DCB) experiments were performed using simulated humid flue gas. Larger quantities of material were needed for these breakthrough measurements (>0.5 g), so MeTBAP-α was used because it was more synthetically accessible.

A range of experiments was performed at different CO_2_ concentrations and relative humidities. Initially, dry DCB traces were recorded at three different CO_2_ concentrations (5%, 10% and 15%) at 298 K and 1000 mbar (Figures 6a, S30 and S31). These CO_2_ concentrations were selected since they fall within the ranges observed in typical flue gas streams.^25^ The calculated CO_2_ uptakes from these DCB measurements were consistent with pure CO_2_ isotherms for dry materials at the same partial pressures (Figure 5a), thus validating the DCB method.

**Figure 6:**
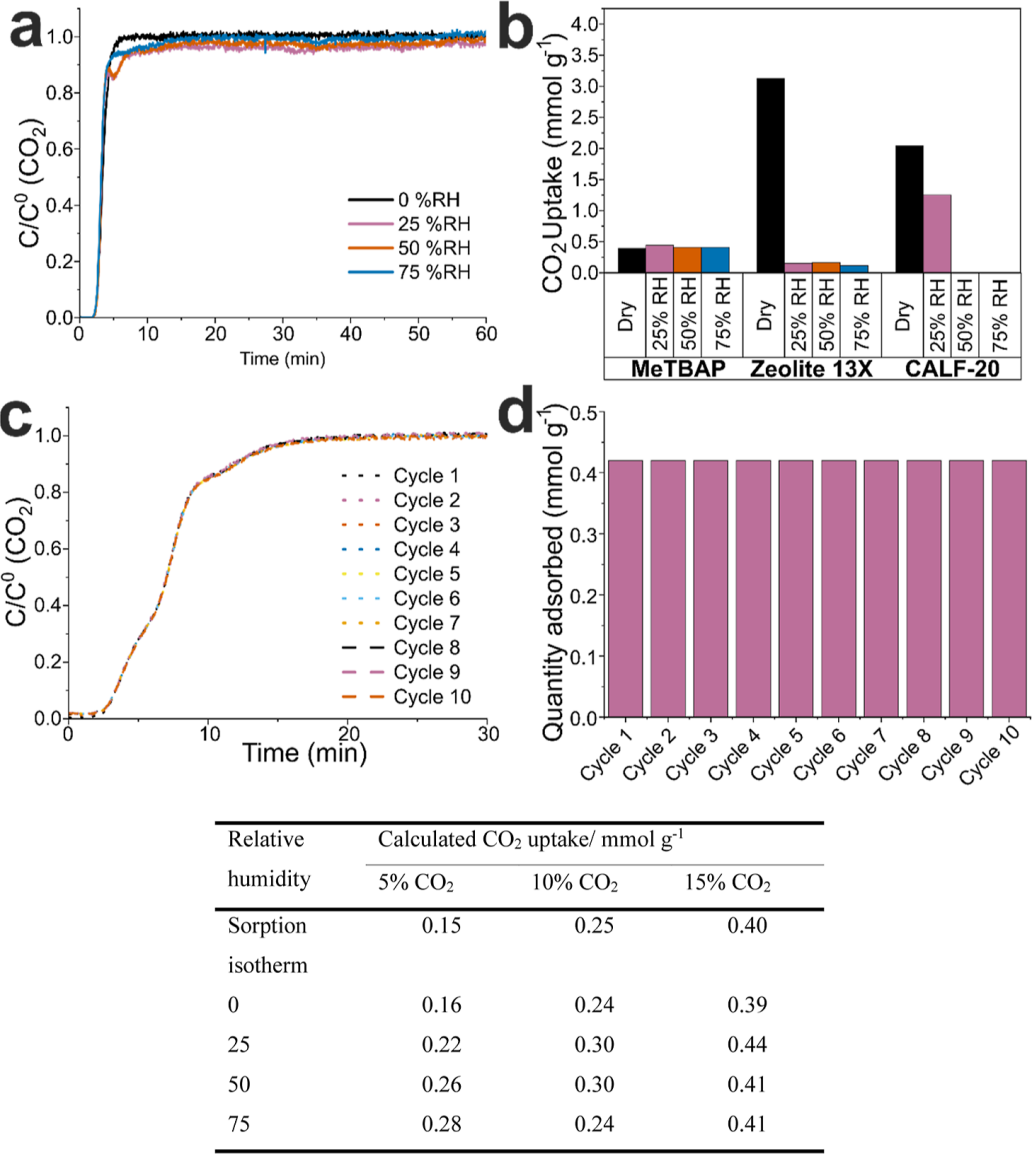
Comparison of CO_2_ separation using MeTBAP-α and two reference frameworks under dry and humid conditions. (a), Dynamic column breakthrough (DCB) traces of normalized flow rate (*F*/*F*_0_) against time for CO_2_ on MeTBAP-α at 298 K using an N_2_:CO_2_ ratio of 85:15 at a range of relative humidities. (b), Comparison of the reduction of CO_2_ capacity for MeTBAP-α, Zeolite 13X and CALF-20 298 K, N_2_:CO_2_ = 85:15, relative humidity = 0–75%; data for MeTBAP-α and Zeolite 13X were measured by us on the same DCB instrument; the data for CALF-20 were extracted from a recent study by Moreton et al in which they measured CO_2_ and water adsorption using mixed gravimetric and conductivity measurements.^30^ that used gravimetric methods. (c) DCB traces of F/F_0_ against time for CO_2_ on MeTBAP-α at 298 K using an N_2_:CO_2_ ratio of 85:15 at 75% RH over ten cycles. (d) bar graph showing the CO_2_ uptakes calculated from the DCB curves for each cycle. Table comparing the CO_2_ uptakes calculated from the breakthrough curves for MeTBAP-α at CO_2_ concentrations of 5, 10 and 15%, as well as the CO_2_ uptake calculated from the CO_2_ sorption isotherm (first row) at 298 K (Figure 2c).

DCB experiments were then performed at the same CO_2_ concentrations at four different relative humidities to determine the effect of water on CO_2_ sorption (Figure 6a). These data demonstrate no reduction in CO_2_ uptake for MeTBAP at relative humidities up to 75%; indeed, a modest increase in CO_2_ capacity was observed, possibly suggesting some weak cooperativity^59–61^ (Figure S36, Section S1.4). Although the CO_2_ capacity for MeTBAP under dry conditions is much lower than zeolite 13X or CALF-20, it shows no decrease in capacity at higher humidities. As such, the working CO_2_ capacity of MeTBAP outperforms both Zeolite 13X and CALF-20 when the relative humidity exceeds 50% (Figure 6b),^30^ which is a more realistic scenario for postcombustion CO_2_ capture from flue gas. MeTBAP is also completely stable to cycling experiments, at least over 10 cycles (Figures 6c–d, S34), showing highly reproducible CO_2_ sorption behavior (Figure 6c) and no drop off in CO_2_ sorption capacity at 75% RH.

To explore the kinetics of the system, the linear driving force model^62^ was used to estimate mass transfer coefficients (*k*_L_) and effective diffusivity (*D*_e_). At all humidities tested we obtained k_L_ values between 12 and 14.4 s^–1^ and *D*_e_ values between 8 × 10^–11^ and 9.6 × 10^–11^ m^2^ s^–1^, showing that humidity has negligible effects on the observed mass transfer kinetics of the system (Figure S33, Section S6).

The direct comparison of DCB data in the literature is complicated by differences in methodology. It can make a large difference whether the sorbent is presaturated with water prior to the breakthrough measurement, as we did here.^63^ The presence of water alters the thermodynamics and kinetics of dynamic CO_2_ adsorption greatly.^64^ Water velocity fronts are well documented to travel much slower than lighter adsorbates in DCB experiments. As such, presaturation is often used to ensure that the entire adsorbent bed is humidified to draw conclusions about materials for CO_2_ capture in humid conditions.^65^ The full regeneration of hydrophilic adsorbents to maintain high CO_2_ performance requires parasitic energy, potentially prohibiting scale-up of processes. For CCS, adsorbents will need to maintain or even increase their working capacities under humid conditions (e.g., ALF,^11^ CALF-20,^28^ IISERP-MOF2,^56^ MUF-17,^66^ Al-PMOF,^46^ *N*,*N*′-dimethylethylenediamine appended Mg-MOF-74,^59,67^ organic macrocycles,^42^ and, here, MeTBAP-α). In this regard, we note that the water uptake in MeTBAP-α (1.3 mmol g^–1^, 298 K, 32 mbar) is lower than all other porous materials summarized in Table S5. Such hydrophobic and water-stable materials might be promising for industrial application, particularly when one considers potential regeneration strategies that involve passing steam over the sorbent.

## 3 Conclusions

Pyrene has been used before as a building block for MOFs with good selectivity for CO_2_ over water.^45^ Pyrene motifs emerged again in an unbiased, diversity-led computational screen of 27,446 molecular fragments as part of our recent study in a search for molecules that combine good CO_2_ binding energies with CO_2_/H_2_O selectivity.^42^ At least three conditions must be satisfied to create porous pyrene-based materials for CCS under moist conditions. First, the pyrene units must be arranged to create a porous framework where the aromatic faces are exposed for CO_2_ sorption. CSP calculations predicted that this would be true for diMeTBAP (Figure 2c,d), but not for its nonmethylated analogue, TBAP, where the pyrene units are π-stacked.^36^ Second, any peripheral, polar functionality, such as carboxylic acids in HOFs, or in MOF linkers, should not affect the CO_2_ selectivity; that is, the pores should be, as much as possible, “pure pyrene” in nature. CSP showed that this would be realized in diMeTBAP-α (and by inference, MeTBAP-α), where the carboxylic acid groups are buried between the 2-D pore layers (Figure 2e), but not for TBAP, where the hydrogen-bonded carboxylic acids line the 1-D pore channels. This was reflected by the extremely low experimental water uptakes for diMeTBAP-α and its isostructural analogue, MeTBAP-α (1.85 and 1.30 mmol g^–1^) compared to TBAP-α (14.24 mmol g^–1^), where the larger pores also allow water condensation. The third condition that must be satisfied is chemical stability under the moist, acidic conditions presented by CCS. Again, crystal energy predictions suggested that the 2-D porous packing for diMeTBAP-α was the thermodynamically most stable crystal packing (Figure 2c), quite unlike TBAP-α, which was a metastable form, predicted to be 57 kJ mol^–1^ less stable than dense, nonporous forms.^35^ These stability predictions are also validated by experiment: for example, the isostructural analogue of diMeTBAP-α, MeTBAP-α, can be boiled in 1 M aqueous acid overnight while retaining its crystallinity and porosity.

Although the dry CO_2_ uptakes for diMeTBAP-α and MeTBAP-α are modest compared to many extended porous frameworks, these HOFs nonetheless outperform benchmark materials such as Zeolite 13X and CALF-20 at relative humidities above 50% (Figure 6d), which are realistic conditions for CCS from industrial flue gas. We ascribe this to the ultrahydrophobic nature of these HOFs (Figure 2g), suggesting that these frameworks or related, more scalable organic materials might be amenable to CCS regeneration strategies such as steam stripping to remove the physisorbed CO_2_.

## Supplementary Material





## Data Availability

All other relevant data generated and analyzed during this study, which include experimental, spectroscopic, crystallographic and computational data, are included in this article and its Supporting Information. The full database of CSP structures have been deposited in cif format in the Zenodo database (10.5281/zenodo.15100686).

## Notes

This document includes full experimental, computational and synthetic methods; and extended NMR, single crystal TGA, gas sorption and dynamic column breakthrough data (PDF)
The associated lattice energies (for all structures) and other predicted properties (for low energy structures) can be found in the file diMeTBAP_Polymorph_Analysis.xlsx (XLSX)
