# Supplementary material for: Selective CO2 Adsorption in Ultrahydrophobic Molecular Pyrene Frameworks by Computational Design
Source: J Am Chem Soc. 2025 Jun 20;147(26):23160–9. doi: 10.1021/jacs.5c06861 (PMC12232307; doi:10.1021/jacs.5c06861)
Supplement: Supplementary file 1 [file ja5c06861_si_001.pdf]

Supplementary Information for

Selective CO_2 Adsorption in Ultrahydrophobic Molecular Pyrene Frameworks by Computational Design

Sam D. Harding[1,†], Tao Liu[1,3,†], Linjiang Chen[1], Siyuan Yang[1], Isaiah Borne[1,3], Thomas Fellowes[1,3], Aaron W. Peters[2], Simon C. Weston[2], John W. Ward[1,3], and Andrew I. Cooper[1,*]

[1]Materials Innovation Factory, Department of Chemistry, University of Liverpool

[2]ExxonMobil Technology and Engineering Company

[3]Leverhulme Research Centre for Functional Materials Design, University of Liverpool

Email: aicooper@liverpool.ac.uk

[†] Equal contributions

Contents

Supplementary Information for 1
Selective CO_2 Adsorption in Ultrahydrophobic Molecular Pyrene Frameworks by 1
Computational Design 1
S1. Computational methods 3
S1.1 Molecular fragment screening 3
S1.2 Crystal structure prediction 3
S1.3 Energy-structure-function map 4
S1.4 Exploring possible cooperative effects 5
S1.5 Electrostatic potential in MeTBAP-α 7
S2. Experimental methods 7
S2.1 Materials 7
S2.2 NMR 7
S2.3 Thermal gravimetric analysis (TGA) 7
S2.4 Single crystal X-ray diffraction (SCXRD) 7
S2.5 Gas sorption analysis 8
S2.6 Powder X-ray diffraction (PXRD) 8
S2.7 Dynamic column breakthrough (DCB) measurements 8

S3. Synthetic Methods 9
S3.1 Synthesis of methyl 3,5-dimethyl-4-(4,4,5,5-tetramethyl-1,3,2- dioxaborolan-2-yl)benzoate 9
S3.2 Synthesis of (4-methoxycarbonyl-2,6-dimethylphenyl)-boronic acid 11
S3.3 Synthesis of 1,3,6,8-tetrakis(methyl 2,6-dimethyl-4-benzoate)pyrene 11
S3.4 Synthesis of 1,3,6,8-tetrakis(2,6-dimethyl-4-benzoic acid)pyrene 11
S3.5 Synthesis of 1,3,6,8-tetrakis(methyl 2-methyl-4-benzoate)-pyrene 13
S3.6 Synthesis of 1,3,6,8-tetrakis(2-methyl-4-benzoic acid)-pyrene 13
S3.7 Crystallisation experiments 13
S3.8 Single crystal preparation 14
S3.9 Stability tests 14
S4. Characterisation Data 14
S4.1 NMR spectroscopy 14
S4.2 SCXRD 19
S4.3 Crystal structure of MeTBAP-α 20
S4.4 Comparison of the crystal structures of TBAP-α, diMeTBAP-α and MeTBAP-α 22
S4.5 Thermogravimetric analysis 23
S5. Gas sorption data 25
S5.1 Nitrogen isotherm for MeTBAP-α after acid treatment (HCl) 25
S5.2 Nitrogen isotherm for MeTBAP-α at 298 K 25
S5.3 CO_2 isotherms for HOFs at 195, 263, 283, 313 and 333 K 26
S5.4 Heat of adsorption data 29
S5.5 Pore Volumes 29
S6. Additional dynamic column breakthrough (DCB) data 30
S7. Performance comparisons with other frameworks in the literature 33
S7.1 Comparison of CO_2-H_2O selectivity (separate isotherms) 33
S7.2 Comparison of wet versus dry CO_2 uptake in breakthrough experiments 35
S7.3 Comparison of Binding Energies 36
S8. References 37

S1. Computational methods

S1.1 Molecular fragment screening

The CHN dataset (9,614 entries) comprises carbon, hydrogen, and nitrogen elements exclusively, and was extracted from Reaxys.[1,2] The molecules were chosen according to specific criteria, including molecular weight < 500 g mol-1, number of elements (<= 3), charge (=0), number of rotational bonds (<=4), and availability from commercial suppliers, both to rationalize the size of the dataset and to bias it toward subsequent experimental accessibility. Three additional datasets, named CHO (12,957 entries), CHF (344 entries), and CHNF (2,837 entries), were extracted using the same criteria.

A computational workflow was developed to screen the binding energies of 27,446 molecular fragments with CO_2 and with H_2O in the gas phase, and hence to assess the likely inherent CO_2/H_2O selectivity of the fragments at low coverage. Briefly, a sampling method was created to calculate the binding energy between CO_2/H_2O and the fragment molecule (here, pyrene) in five steps, initially by using Self-Consistent Tight-Binding (TB) method implemented in xTB[3,4] for a fast structure screening followed by a DFT[5,6] method at B97D3[7,8]/Def2-SVP[9] level with dispersion[10], by considering both accuracy and efficiency, for energy correction using Gaussian16[11]. The full details of this method, and a discussion of other fragments arising from the computational screen, are given in ref. 42 in the main text.

S1.2 Crystal structure prediction

The molecular geometry of diMeTBAP was fully optimized using the Dmol3[12] module, within BIOVIA Materials Studio[13]. The generalized gradient approximation (GGA) with the Perdew–Burke–Ernzerhof (PBE[14]) exchange-correlation functional was employed to treat electron interaction energy, with Grimme's dispersion correction. The basis set was defined as DNP. After optimization, electrostatic-potential-fitted charges were calculated and assigned to diMeTBAP's atoms. The total charge of the molecule was set to 0. These atomic charges were used together with the DREIDING[15] forcefield in the subsequent conformer search and crystal structure prediction (CSP) calculations.

Conformer searching yielded only one conformer, with all four 2,6-dimethylphenyl units perpendicular to the pyrene core, disregarding the rotation of each carboxylic acid with respect to its attaching 2,6-dimethylphenyl unit. Conformers differing only in the carboxylic acid orientation yielded negligible differences in energy; we therefore chose the conformer with the highest molecular symmetry for subsequent CSP calculations.

For CSP, the Polymorph module in Materials Studio was employed to perform crystal structure predictions based on the molecular configuration determined in the previous step, which remained rigid throughout all CSP calculations. Only this single conformer was considered in CSP. Trial crystal structures were generated with one molecule in the asymmetric unit for the 10 most common space

groups: P21/C, P-1, P212121, C2/C, P21, PBCA, PNA21, CC, PBCN, and C2. Duplicate structures were removed using the same module, with the maximum clusters set to 2000.

S1.3 Energy-structure-function map

The CO_2 adsorption capacities of the 3,384 lowest energy crystal structures of diMeTBAP were calculated using Grand-Canonical Monte Carlo (GCMC) method[16] and the simulations were performed with the RASPA code.[17] In the GCMC simulation, the chemical potential, volume, and temperature were kept fixed while the number of gas molecules in the adsorbed phase is allowed to fluctuate so that the chemical potentials of the adsorbed phase and the bulk gas reservoir are equal. The GCMC simulations performed in this work included a 100,000-cycle equilibration period and a 200,000-cycle production run. The trial MC moves including insertion, deletion, translation, rotation, and reinsertion were randomly selected with equal probabilities.

The atomistic representation of 3,384 diMeTBAP crystal structures were constructed from the CSP crystallographic data, with all the atoms kept fixed at their positions during the simulation. In order to ensure statistical meaningfulness and numerical accuracy, a × b × c unit-cell representations of the diMeTBAP crystal structure was used in the GCMC simulations, a/b/c are the repeating unit in three dimensions. Specific a × b × c was applied to each crystal structure to make sure the half length of the cell a/b/c is larger than the energy cut off. The host–guest and guest–guest interaction energies were determined according to the Lennard–Jones (LJ) and Coulomb potentials. A cut-off radius of 12.0 Å was used for all LJ interactions (shifted), while all Coulomb interactions were computed using the Ewald summation technique with a relative precision of 10^{-6}. The Lorentz–Berthelot combining rules were used to calculate the LJ cross-parameters.

The force-field parameters used for the adsorbates were taken from the literature and are summarized in Table S1. The LJ parameters for the diMeTBAP atoms were assigned based on the DREIDING force field.[13] To obtain partial atomic charges for the diMeTBAP host, chargemol[18] was used to fit density-derived electrostatic and chemical charge method (DDEC)[19,20] by using the cube files obtained by CP2K[21].

Table S1: **Force-field parameters for diMeTBAP and CO_2 in GCMC.**

	Atom type	e/k_B (K)	s (A)	q (e)
Carbon dioxide[22]	O (CO_2)	79.000	3.050	-0.350
	C (CO_2)	27.000	2.800	0.700
diMeTBAP	C (diMeTBAP)	47.8562	3.47299	DDEC6
	H (diMeTBAP)	7.648938	2.84642	DDEC6
	O (diMeTBAP)	48.1581	3.03315	DDEC6

The calculated CO_2 adsorption capacities were then used to plot an energy-structure-function map (main text, Figure 2c).

S1.4 Exploring possible cooperative effects

There was some evidence for a small increase in CO_2 uptake in MeTBAP-α at higher relative humidities (Supplementary Figure S27). We therefore explored the potential origin of this cooperative effect *in silico*. We did this for the isomorphous analogue of MeTBAP-α, diMeTBAP-α, because the lack of isomers in diMeTBAP made it simpler to construct unambiguous periodic cells for the simulations. Based on experimentally determined adsorption capacities, a maximum of three water molecules can be accommodated in one pore in the simulation cell at 99% relative humidity (RH). Similarly, in simulated flue gas containing 5% CO_2, up to six CO_2 molecules can be present in the same pore. We employed the Docking Submodule (aISS)[23] implemented in xTB[24] by extracting a cluster model of a single pore from the crystal structure comprising four diMeTBAP molecules. This model was then loaded with varying quantities of water molecules (ranging from 0 to 4) to simulate different RH conditions. Subsequently, we introduced 1–6 CO_2 molecules into each RH scenario, resulting in a total of 30 distinct models, including dry conditions (no water).

To maintain consistency with the crystal structure, we kept the diMeTBAP framework fixed, allowing only CO_2 and H_2O molecules to relax within the pore cavity. Afterwards, B97D3/DFT method with D3 version of Grimme's dispersion implemented was used to correct the energy. The results of these simulations are shown in Figure S2.

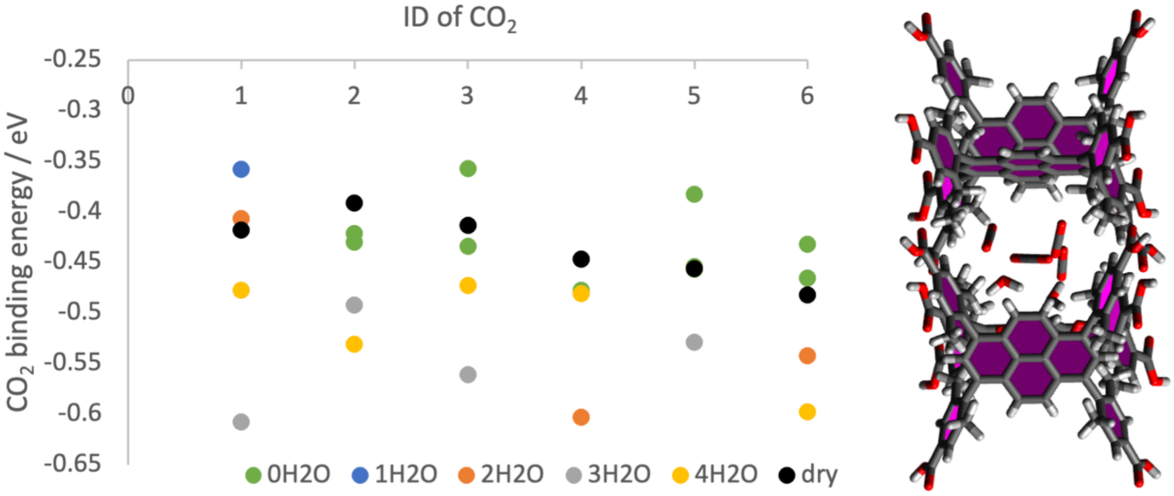

Figure S2. Calculated CO_2 binding energies in diMeTBAP-α at different water levels. Black points indicate dry conditions, while green points represent CO_2 molecules that do not interact with any water molecules under wet conditions. Other coloured points show CO_2 molecules that interact with one or

more water molecules in the wet condition. The structure on the right illustrates a simulated example of the humid condition, featuring 3 H_2O molecules and 5 CO_2 molecules within the pore.

Supplementary Figure S2 presents the binding energy of each CO_2 molecule as it is introduced into the model. Black data points indicate CO_2 interactions exclusively with the diMeTBAP framework under dry conditions, while green data points represent interactions solely with the diMeTBAP framework under wet conditions. Data points in other colors show cases where CO_2 interacts with one or more water molecules under wet conditions, regardless of whether it also interacts with the diMeTBAP framework.

In general, the calculated binding energy of CO_2 under 'wet' conditions (CO_2 interacts with one or more water represented by blue, orange, grey, and yellow data points) is more negative (stronger) than under dry conditions (black data points). In addition, if CO_2 interacts with framework only (green data points) at wet condition, it has similar binding energy as at dry condition. Furthermore, the average of the six CO_2 molecules' binding energies increases steadily from -0.43 to -0.51 eV when going from dry conditions to 4 water molecules in the pore. These simulations agree qualitatively with experiment, suggesting a stronger average binding of CO_2 under humid conditions. These results agree with previous work,[25] indicating that hydrogen bonding between water molecules clustered around carbonyl groups might play a role in stabilising CO_2 binding.

S1.5 Electrostatic potential in MeTBAP-α

A five-molecule cluster was extracted from the single crystal structure geometry for MeTBAP-α, which was then optimised (GFN2-xTB[3,4]). A single point calculation was performed at the PBE[14]/def2-SVP[9] level to afford the electron density. The molecular electrostatic potential was visualised on the 0.005 a.u. electron density isosurface, ranging from -0.2 a.u. to 0.2 a.u (Fig. 2g, main text).

S2. Experimental methods

S2.1 Materials

Methyl 3-methyl-4-(4,4,5,5-tetramethyl-1,3,2-dioxaborolan-2-yl)benzoate, methyl 4-bromo-3,5-dimethylbenzoate, Pd(dppf)Cl_2, bispinacolato diboron, potassium acetate, 1,3,6,8-tetrabromopyrene were purchased from Fluorochem. Solvents and acids purchased from Fisher. Anhydrous solvents were purchased from Sigma-Aldrich. All chemicals were used without further purification. All gases for sorption analysis were supplied by BOC at a purity of ≥99.9%. Reactions were carried out under a N_2 atmosphere using standard Schlenk techniques.

S2.2 NMR

NMR spectra were recorded on a Bruker Advance 400 NMR spectrometer at 400.13 MHz (1H) and 100.61 MHz (13C). ^{13}C spectra are ^{1}H decoupled.

S2.3 Thermal gravimetric analysis (TGA)

TGA analysis was carried out using a TA Q5000 IR analyzer with an automated vertical overhead thermobalance. Samples were heated under nitrogen at a rate of 10 °C min^{-1} up to 900 °C.

S2.4 Single crystal X-ray diffraction (SCXRD)

Single crystals of MeTBAP-α were grown by vapour diffusion of chloroform into a DMF solution, affording colourless, almost spherical polyhedra. A suitable crystal was harvested in a drop of mother liquor, and mounted on a Mitegen loop. This was immediately transferred to the diffractometer and placed under an open flow helium cooling device (Oxford Cryosystems Helix) at 30 K. Intensity data were collected on beamline I19 (EH1) at Diamond Light Source (Didcot, UK) using silicon double crystal monochromated synchrotron radiation (λ = 0.6889 Å) and a Dectris Pilatus 2M detector. Data were collected using a combination of ω and ϕ scans, on a 3-circle (fixed κ) goniometer. Indexing, integration and absorption corrections were performed using the xia2 automated pipeline. The structure was solved using SHELXT[26]and refined by full-matrix least-squares on $|F^2|$ using SHELXL,[27] integrated within Olex2.[28] All hydrogen atoms were placed geometrically and refined using a riding model. All heavy atoms were refined anisotropically. A solvent mask was used to remove the scattering contribution of disordered solvent voids. For full refinement details, see section S5.3.

S2.5 Gas sorption analysis

All gas sorption measurements were performed using a Micromeritics 3flex surface characterization analyzer. Samples were degassed on the analysis port at 100 °C under vacuum of 10-4 mbar. During analysis, the temperature was controlled using a ColdEdge Technologies liquid He cryostat chiller unit. Surface areas were measured by N_2 sorption at 77 K. Apparent Brunauer–Emmett–Teller (BET) surface areas for TBAP-α, MeTBAP-α and diMeTBAP-α were calculated using data in a relative pressure range chosen to fulfil best the criteria proposed by Rouquerol et al.[31]

S2.6 Powder X-ray diffraction (PXRD)

PXRD patterns for TBAP-α, MeTBAP-α and diMeTBAP-α were collected in vertical transmission mode from loose powder samples held on Mylar film in aluminium well plates, using a Panalytical Empyrean equipped with a high throughput screening XYZ stage, X-ray focusing mirror and PIXcel detector with Cu-Kα radiation (λ = 1.541 Å).

Simulated PXRD patterns were refined against experimental data GSASii.[32]

High-resolution variable temperature PXRD (VT-PXRD) of MeTBAP-α was conducted at the I11 beamline of the Diamond Light Source using position-sensitive detectors (PSD) and monochromated radiation (λ = 0.823918 Å). The MeTBAP-α sample was loaded into capillary tubes of 0.7 mm diameter and flame sealed. VT-PXRD data of MeTBAP-α were then collected using a heating rate of 300°C·hr−1 from 25°C to 800°C. One minute was given for the temperature of the HOF to stabilise after each heating process.

High-resolution gas loading PXRD (GL-PXRD) of MeTBAP-α was conducted at the I11 beamline of the Diamond Light Source using position-sensitive detectors (PSD) and monochromated radiation (λ = 0.823918 Å). The MeTBAP-α sample was loaded into a capillary tube of 0.7 mm diameter, mounted on a gas loading cell equipped with rocking stage and loaded at with CO_2 between 0.1 and 10 bar at 298 K. The sample was reactivated between different gas loadings.

S2.7 Dynamic column breakthrough (DCB) measurements

Dynamic column breakthrough (DCB) traces were collected using a Hiden Isochema ABR automated breakthrough analyser. Column breakthrough experiments were run at a controlled temperature using water bath or furnace between 25–90 °C and a steady post-column pressure of 1000 mbar. A mass spectrometer (Hiden Analytical DSMS) was placed at the outlet to record the composition of the effluent gas. The instrument was connected to a vapour generator set between 25- 50 °C. The equipment schematic is shown in Figure S2. A 2 mL stainless steel column (length 10 cm; inner diameter 0.5 cm) was packed with analyte (0.1-1 g), glass wool was then added to both ends of the column to prevent any contamination to the system. Data were recorded at 3 second intervals using Isochema HIsorp 2017 software. The column was activated at 90 °C for 12 hours using helium as a purge gas prior to each

experiment. **<u>Before recording CO_2 breakthrough under humid conditions, the column was always exposed to nitrogen at the desired relative humidity until saturated.</u>**

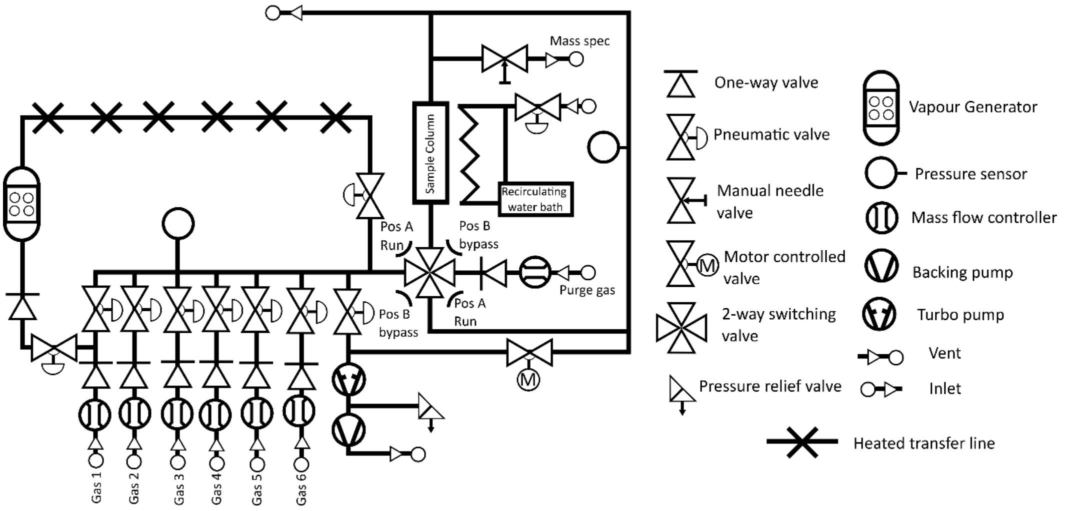

Figure S3. Schematic diagram of the breakthrough apparatus.

Gas adsorption capacities were then calculated using the following equation:

$$\int_0^{t_\infty}\left(1-\frac{F}{F_0}\right)dt_{sample} - \int_0^{t_\infty}\left(1-\frac{F}{F_0}\right)dt_{blank} = \frac{V\varepsilon}{Q_0}\left(1+\frac{1-\varepsilon}{\varepsilon}\frac{q_{ads}}{c_{avg}}\right)$$

Where F and F_0 are the outlet and inlet flow rate of the adsorbate, respectively, V is the volume of the column, ε is the void space fraction, Q_0 is the total volumetric flow rate at the inlet, c_{avg} is the average adsorbate concentration across the column and q_{ads} is the amount of adsorbate in the adsorbed phase at equilibrium. Adapted from Wilkins *et al.*[33]

Breakthrough kinetics were analysed using the linear driving force model.[34] The effective mass transfer coefficient (k_L) was calculated by fitting the breakthrough curves using the following equation:

$$\frac{c}{c_0} = 1 - e^{-k_L t}$$

To calculate the effective diffusivity (D_e), particles were assumed to be spheres of average radius (R) 10 µm and the following equation was used:

$$k_L = \frac{15D_e}{R^2}$$

S3. Synthetic Methods

S3.1 Synthesis of methyl 3,5-dimethyl-4-(4,4,5,5-tetramethyl-1,3,2- dioxaborolan-2-yl)benzoate

Under nitrogen, a flask was charged with methyl 4-bromo-3,5-dimethyl-benzoate (4.8 g, 20 mmol), bispinacolato diboron (4.8 g, 19 mmol), potassium acetate (4.8 g, 50 mmol) and Pd(dppf)Cl2 (500 mg, 0.7 mmol). Dry dioxane (50 mL) was added and the suspension was heated to 90 °C for 3 days. The reaction was allowed to cool and 1 M HCl (100 mL) was added and extracted with ethyl acetate (3 x 100 mL). The organic layer was dried over magnesium sulfate and the solvent was removed under reduced pressure. The resulting solids were then separated by column chromatography to yield the product as a colourless crystalline solid (3.98 g, 70%); δ ^{1}H NMR/ppm (400 MHz, CDCl3) 7.61 (2 H, s, ArH), 3.88 (3 H, s, OCH 2), 2.43 (6 H, s, ArCH 3), 1.42 (12 H, s, pinCH 3).

S3.2 Synthesis of (4-methoxycarbonyl-2,6-dimethylphenyl)-boronic acid

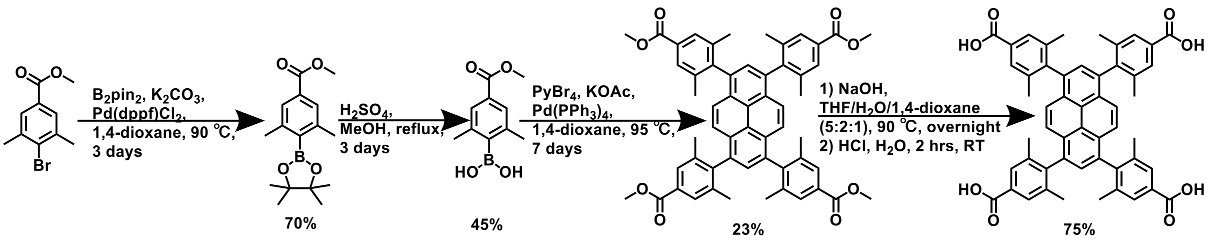

Figure S4. Synthetic route to diMeTBAP

3,5-dimethyl-4-(4,4,5,5-tetramethyl- 1,3,2-dioxaborolan-2-yl)benzoate (3.56 g, 12 mmol) was suspended in a mixture of methanol (40 mL) and H_2SO_4 (12 M, 5.4 mL) and refluxed for 72 hours. The mixture was then cooled, neutralised with potassium carbonate and the solvents removed under reduced pressure. The solids were then suspended in water (10 mL) and extracted with ethyl acetate (5 x 10 mL). The organic phase was then dried over magnesium sulfate and the solvents removed under reduced pressure. This solid residue was then rinsed with the minimum amount of ice-cold ethyl acetate to remove starting material to yield the product as a white powder (1.14 g, 45%); δ ^{1}H NMR/ppm (400 MHz, DMSO) 7.54 (2 H, s, ArH), 3.82 (6 H, s, OCH 2), 2.33 (6 H, s, ArCH 3).

S3.3 Synthesis of 1,3,6,8-tetrakis(methyl 2,6-dimethyl-4-benzoate)pyrene

Under nitrogen, a flask was charged with (4-methoxycarbonyl-2,6-dimethylphenyl)boronic acid (1 g, 4.8 mmol), 1,3,6,8-tetrabromopyrene (0.41 g g, 0.8 mmol), potassium carbonate (0.6 g, 6 mmol), and $Pd(PPh_3)_4$ catalyst (100 mg, 0.1 mmol). Dry dioxane (25 mL) was added and the suspension was heated to 90 °C for 7 days. The reaction was allowed to cool and 1 M HCl (100 mL) was added and extracted with chloroform (3 x 100 mL). The organic layer was dried over magnesium sulfate and the solvent was removed under reduced pressure. The resulting solids were then separated by column chromatography to yield (0.199 g, 23 %); δ ^{1}H NMR/ppm (400 MHz, CDCl3) 7.91 (8 H, s, ArH), 7.60 (2 H, s, PyH), 7.51 (4 H, s, PyH), 3.98 (3 H, s, COOCH 3), 2.05 (24 H, s, ArCH 3).

S3.4 Synthesis of 1,3,6,8-tetrakis(2,6-dimethyl-4-benzoic acid)pyrene

1,3,6,8-tetrakis(methyl 2-methyl-4-benzoate)pyrene was suspended in a mixture of water, dioxane and THF (2:2:5, 10 mL), aqueous NaOH (saturated, 1 mL) was added and the mixture was stirred overnight at 90 °C. The resulting mixture was neutralised using HCl (1 M, 5 mL) and the solvents removed under reduced pressure. The resulting residue was suspended in water and stirred at room temperature for 2 hours before acidifying with HCl (1 M, 10 mL). The solids were filtered off, washed with HCl (1 M, 20 mL), water (50 mL) and diethyl ether (50 mL) and then dried in a vacuum oven at 80 °Covernight

to yield the product as a dark grey powder (0.14 g, yield: 75%); δ ^{1}H NMR/ppm (400 MHz, DMSO) 7.75 (8 H, s, ArH), 7.51 (2 H, s, PyH), 7.37 (4 H, s, PyH), 1.83 (24 H, s, ArCH 3).

S3.5 Synthesis of 1,3,6,8-tetrakis(methyl 2-methyl-4-benzoate)-pyrene

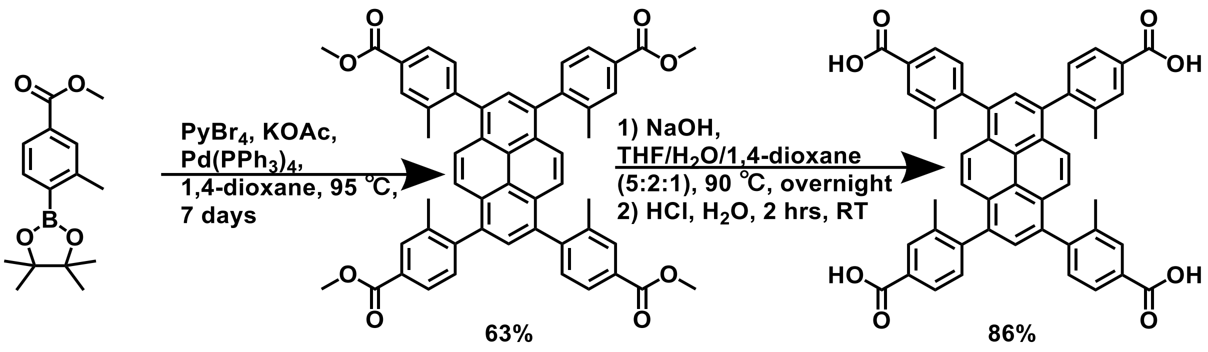

Figure S5. Synthetic route to MeTBAP

Under nitrogen, a flask was charged with 4-methoxycarbonyl-2 -methyl-benzeneboronic acid pinacol ester (4.9 g, 18 mmol), 1,3,6,8-tetrabromopyrene (1.5 g, 3.0 mmol), potassium carbonate (2.7 g, 20 mmol), and Pd(PPh3)4 (100 mg, 0.1 mmol). Dry dioxane (100 mL) was added and the suspension was heated to 90 °C for 7 days. The reaction was allowed to cool and 1 M HCl (100 mL) was added and extracted with chloroform (3 x 100 mL). The organic layer was dried over magnesium sulfate and the solvent was removed under reduced pressure. The resulting solids were then separated by column chromatography to yield (1.48 g, yield: 63%); δ ^{1}H NMR/ppm (400 MHz, CDCl3) 8.07 (4 H, m, ArH), 8.00 (4 H, m, ArH), 7.75 (2 H, m, PyH), 7.66 (4 H, m, PyH), 7.46 (4 H, m, ArH), 3.97 (12 H, m, COOCH 3), 2.15 (12 H, s, ArCH 3).

S3.6 Synthesis of 1,3,6,8-tetrakis(2-methyl-4-benzoic acid)-pyrene

1,3,6,8-tetrakis(methyl 2-methyl-4-enzoate)pyrene was suspended in a mixture of water, dioxane and THF (2:2:5, 50 mL), aqueous NaOH (saturated, 5 mL) was added, and the mixture was stirred overnight at 90 °C. The resulting mixture was neutralised using HCl (1 M, 20 mL) and the solvents 5removed under reduced pressure. The resulting residue was suspended in water and stirred at room temperature for 2 hours before acidifying with HCl (1 M, 10 mL). The solids were filtered off, washed with HCl (1 M, 50 mL), water (100 mL) and diethyl ether (100 mL) and then dried in a vacuum oven at 80 °C overnight to yield the product as an off-white powder (1.14 g, yield 86%); δ ^{1}H NMR/ppm (400 MHz, DMSO) 8.00 (4 H, m, ArH), 7.92(4 H, m, ArH), 7.77(2 H, m, PyH), 7.64 (4 H, m, PyH), 7.51 (4 H, m, ArH), 2.10 (12 H, s, ArCH 3).

S3.7 Crystallisation experiments

A saturated solution of diMeTBAP, MeTBAP or TBAP in DMF (1 mL) in a 4 mL sample tube was placed into a larger vessel containing excess chloroform. In each case, the solution was left several days until precipitation occurred. The solvent was then removed by pipette and the solids washed by solvent exchange with diethyl ether followed by drying under ambient conditions.

S3.8 Single crystal preparation

diMeTBAP or MeTBAP (5 mg) was dissolved in DMF (2.5 mL) in a small sample vial. The vial was then placed into a larger vial containing chloroform and placed in the fridge. Crystals formed by slow infiltration of chloroform into solution. The obtained crystals were desolvated by solvent exchange with diethyl ether followed by drying under ambient conditions.

S3.9 Stability tests

Samples of crystalline MeTBAP-α (20 mg) were placed in sample vials and suspended in 1 mL of either water, 1M HCl, 1M sulfuric acid or 1M nitric acid. Samples were then either left to stand for 3 days or boiled at 100 °C overnight. The solids were then filtered off and washed with water (3 x 4 mL) followed by diethyl ether (3 x 2 mL) and dried in a vacuum oven. PXRD patterns of each sample was then recorded, as described previously.

S4. Characterisation Data

S4.1 NMR spectroscopy

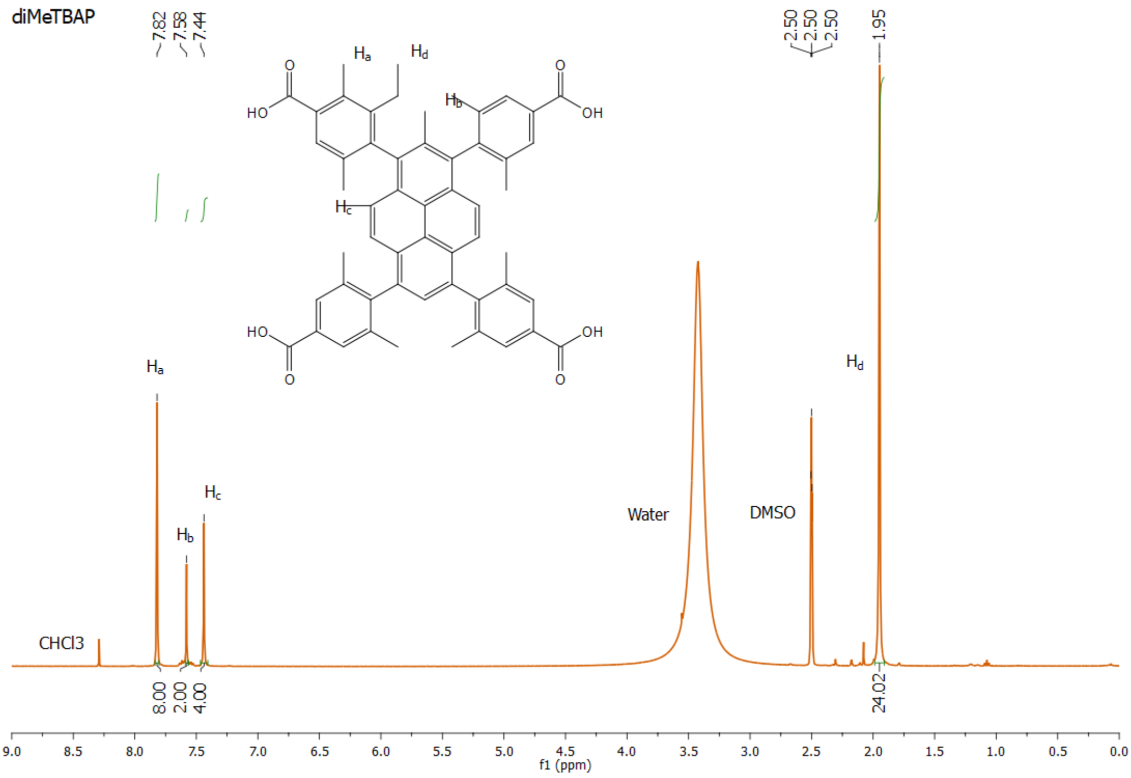

Figure S6. diMeTBAP ^{1}H NMR recorded in DMSO-d6

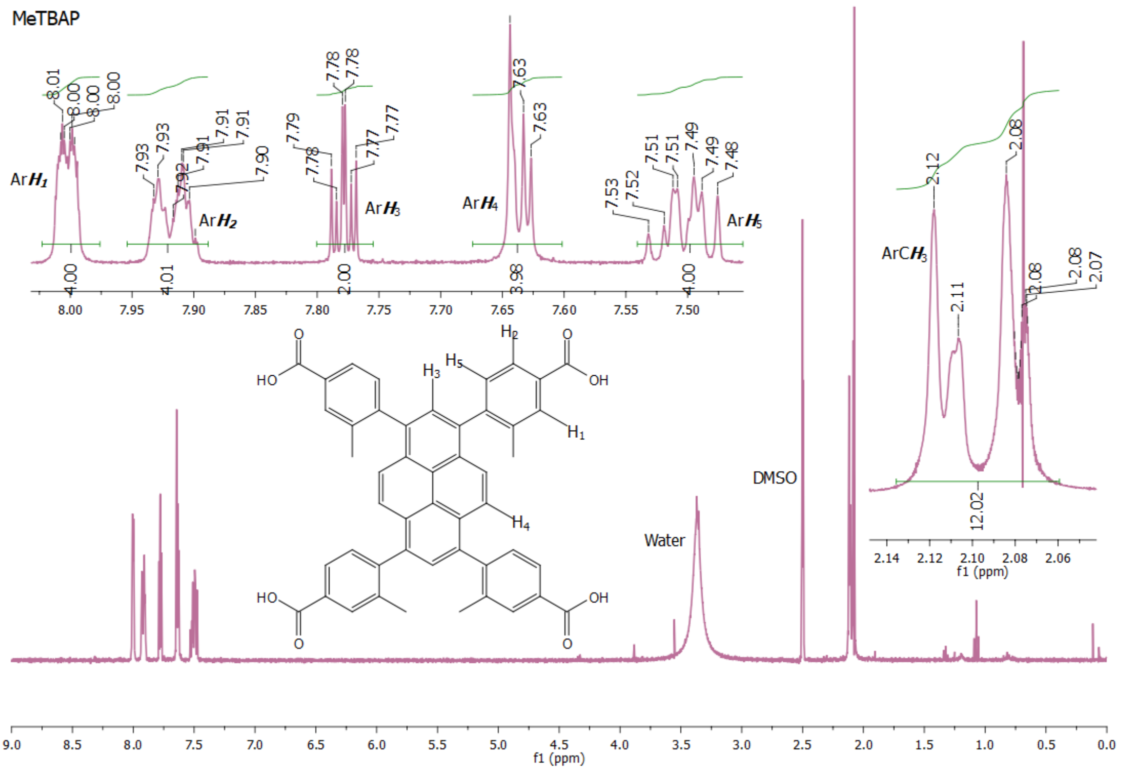

Figure S7. MeTBAP ^{1}H NMR recorded in DMSO-d6

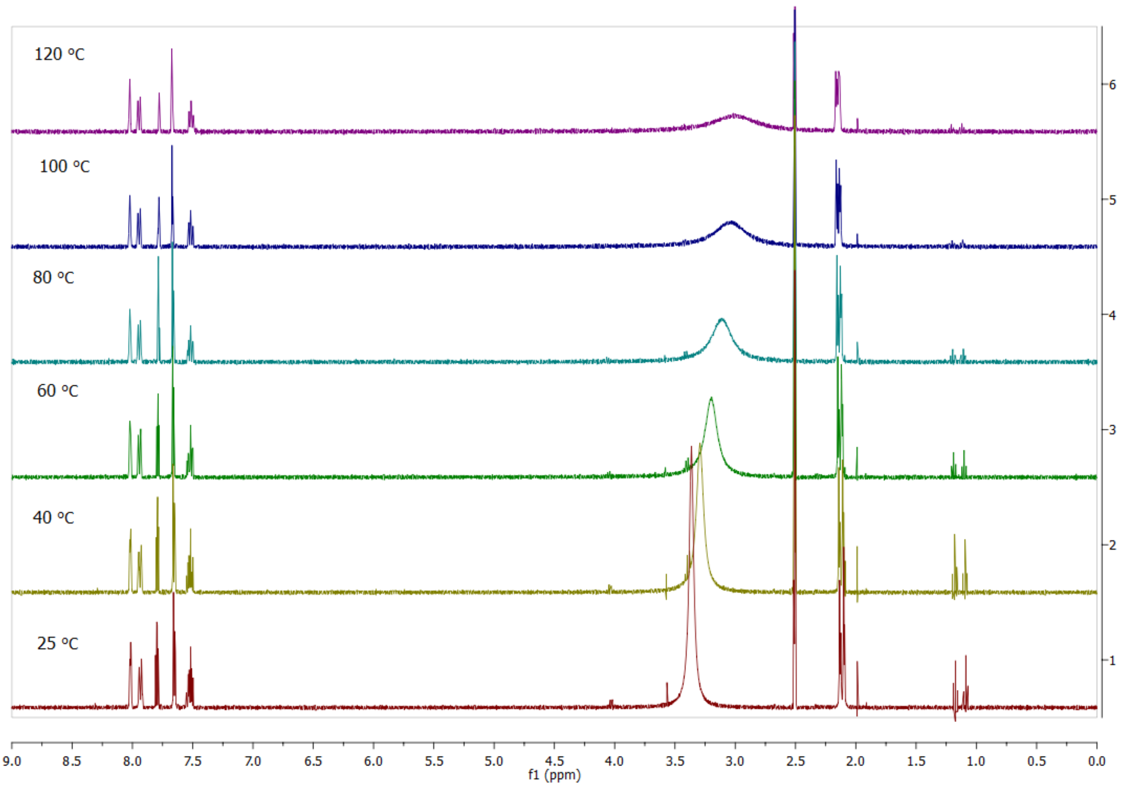

Figure S8. Variable temperature ^{1}H NMR (VT-NMR) of MeTBAP, 25–120 °C recorded in DMSO-d6

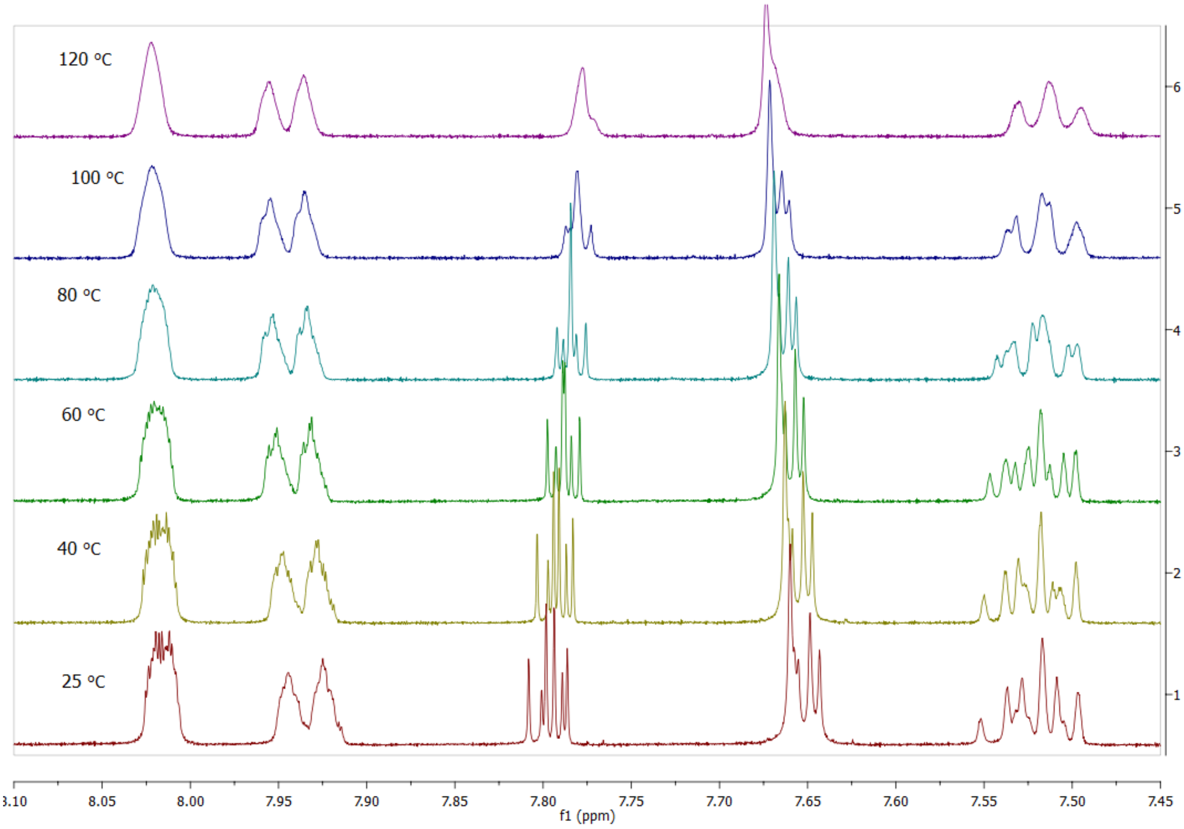

Figure S9. Aromatic region of the ^{1}H VT-NMR of MeTBAP, 25–120 °C recorded in DMSO-d6

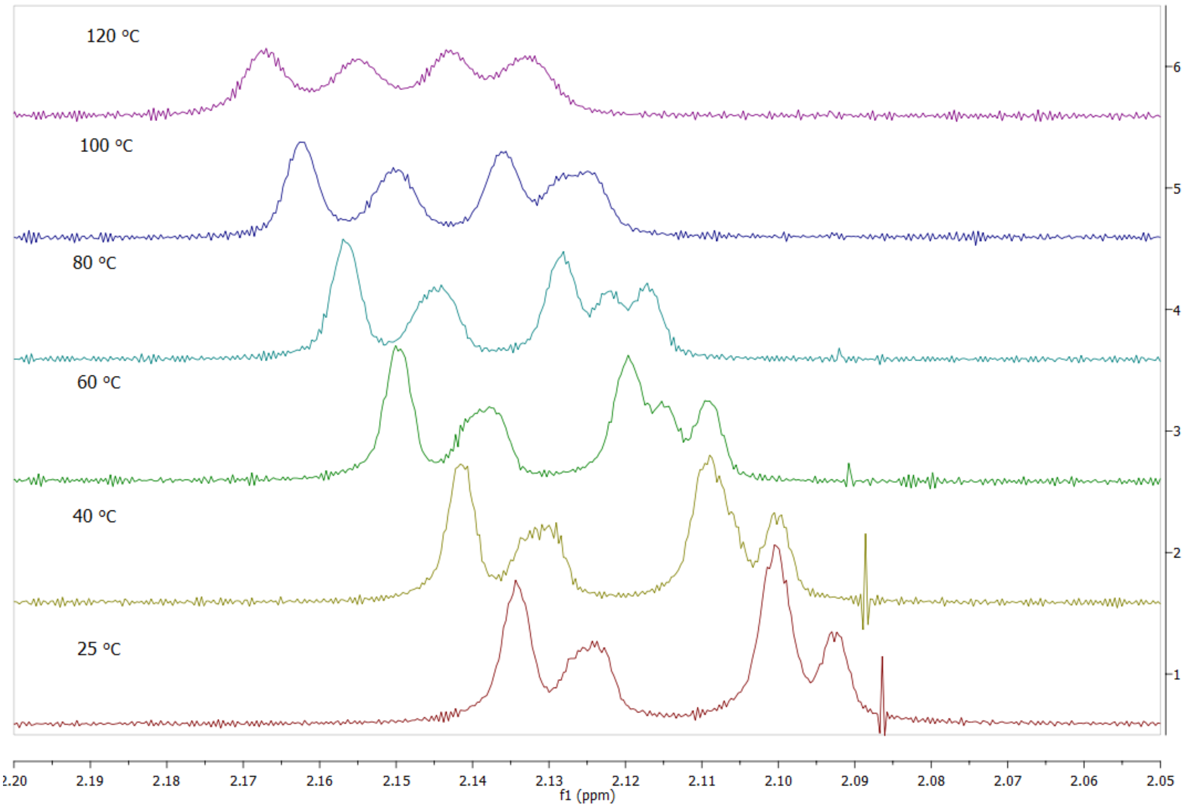

Figure S10. Aliphatic region of the ^{1}H VT-NMR of MeTBAP, 25–120 °C recorded in DMSO-d6

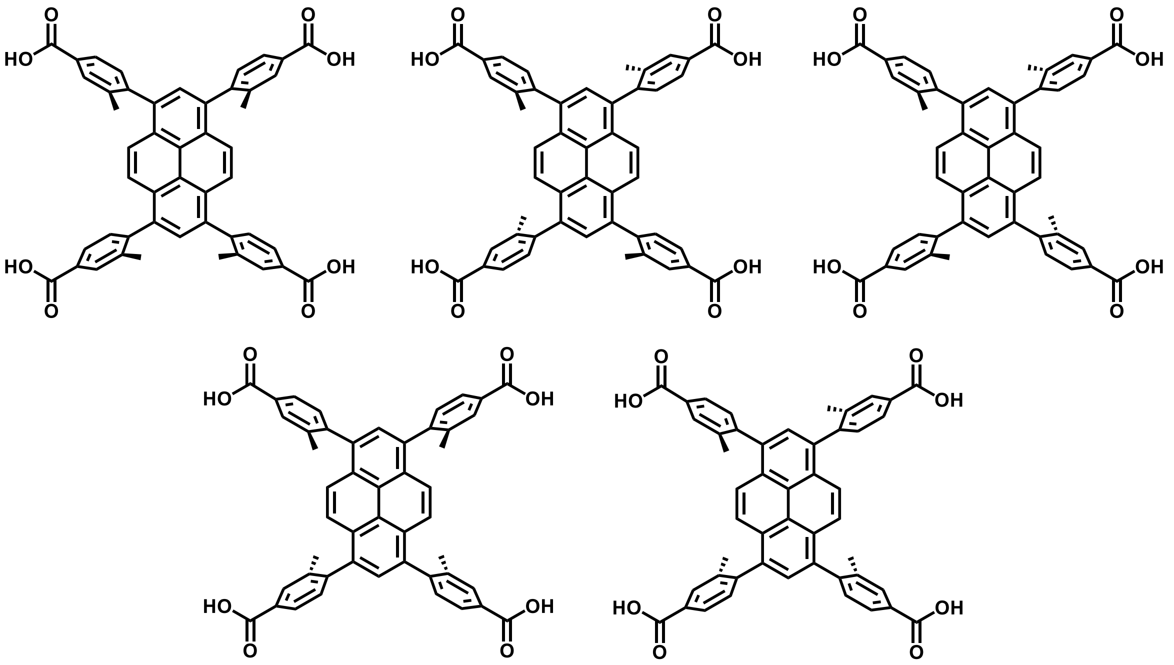

Figure S11: The five possible isomers of MeTBAP (ignoring relative orientation of carboxylic acids).

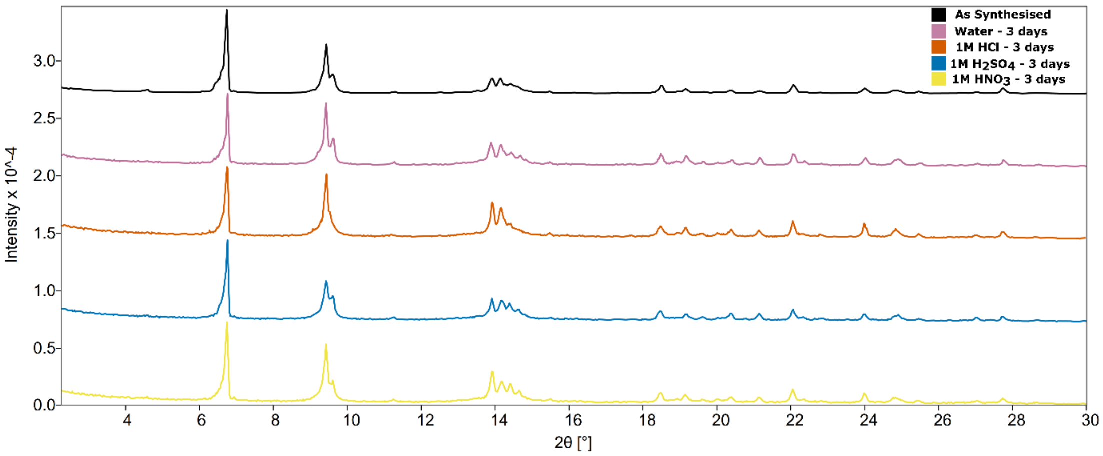

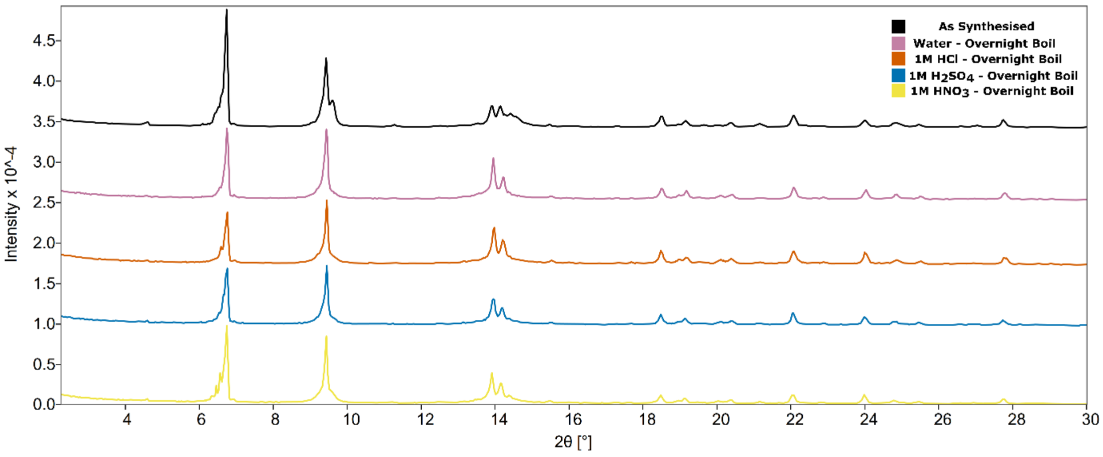

Figure S12. Stability tests for MeTBAP HOF. PXRD of MeTBAP-α after exposure to different aqueous and acidic conditions (upper = room temperature for 3 days, lower = overnight boil).

S4.2 SCXRD

The diffraction data showed significant diffuse scattering even at 30 K, which may be attributed to disorder of the pyrene core and phenyl substituents (evident in the elongated ellipsoids). The data showed no evidence of twinning or other deficiencies.

Table S2. Crystal data and structure refinement for MeTBAP-α.

Identification code	SH_MeTBAP
Empirical formula	$C_{57}H_{55}N_3O_{11}$
Formula weight	958.04
Temperature/K	30.15
Crystal system	orthorhombic
Space group	Cmce
a/Å	25.193(3)
b/Å	13.7483(4)
c/Å	20.2399(11)
a/Å	25.193(3)
b/Å	14.794(3)
c/Å	14.2841(15)
α/°	90
β/°	90
γ/°	90
Volume/Å^3	5323.7(14)
Z	4
ρ_{calc}g/cm^3	1.195
μ/mm^1	0.078
F(000)	2024.0
Crystal size/mm^3	0.15 × 0.15 × 0.13
Radiation	Synchrotron (λ = 0.6889)
2Θ range for data collection/°	4.15 to 45.1
Index ranges	-27 ≤ h ≤ 26, -15 ≤ k ≤ 16, -15 ≤ l ≤ 15
Reflections collected	14456
Independent reflections	1971 [R_{int} = 0.0515, R_{sigma} = 0.0502]
Data/restraints/parameters	1971/0/138
Goodness-of-fit on F^2	1.141

Datablock sh_metbap - ellipsoid plot

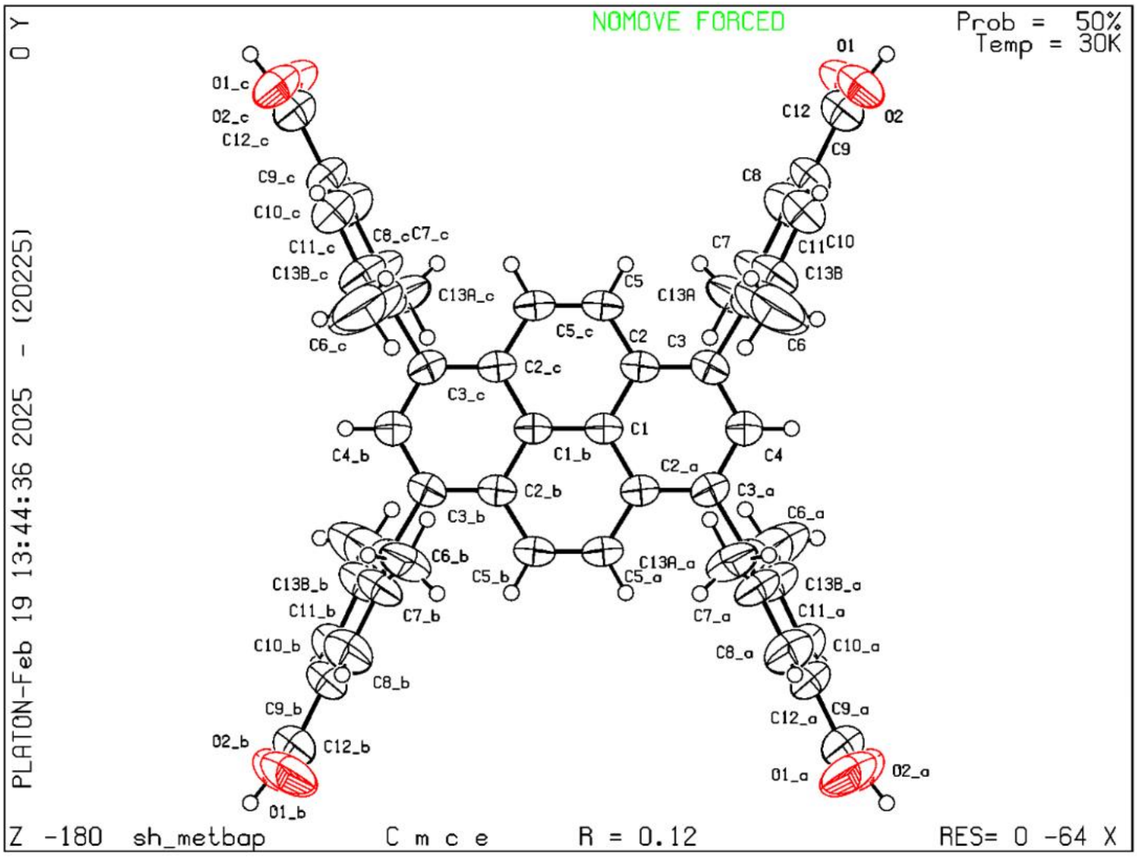

Figure S13. Thermal ellipsoid plot of MeTBAP-α. Ellipsoids are drawn at the 50% probability level.

S4.3 Crystal structure of MeTBAP-α

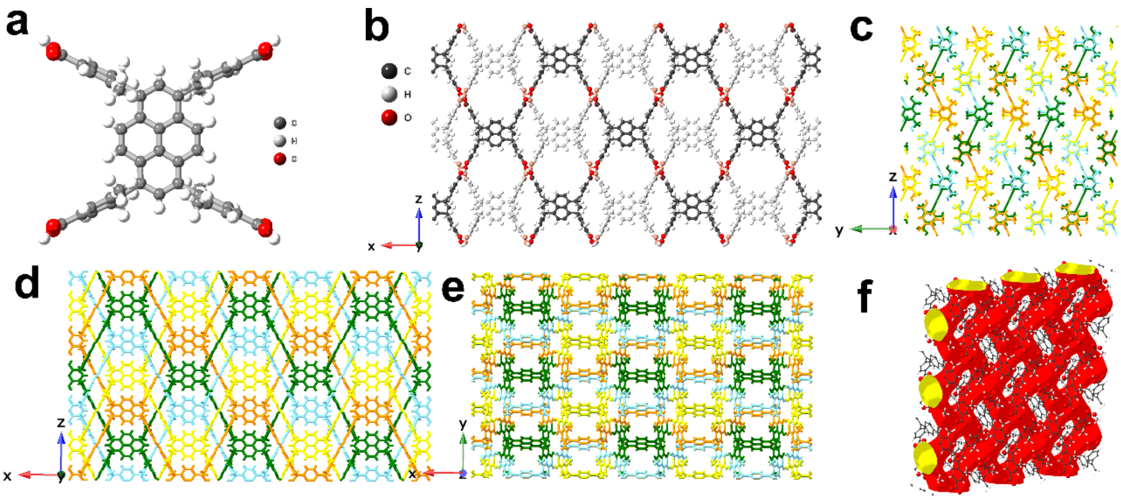

Figure S14. The structure of MeTBAP-α derived from single crystal x-ray diffraction depicting **a**, a single molecule of MeTBAP (due to the disorder in the structure, the molecule appears with methyl groups in both the 3 and 5 positions of the benzoic acid at half occupancy; **b**, AB stacked layers of

MeTBAP; **c**, the interpenetrated structure of MeTBAP viewed along its long axis; **d** and **e**, the interpenetrated structure of MeTBAP viewed along its two short axes; **f**, representation of a single 2-D pore layer in MeTBAP-α (yellow = pore interior).

S4.4 Comparison of the crystal structures of TBAP-α, diMeTBAP-α and MeTBAP-α

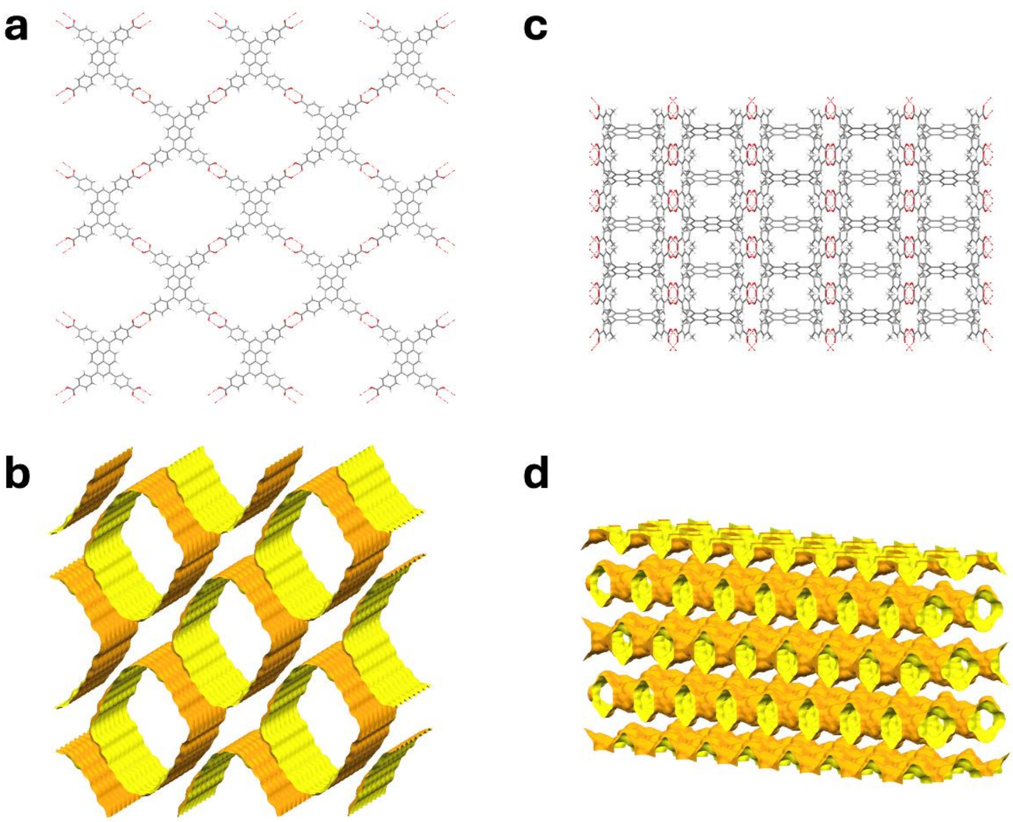

Figure S15. A comparison of the pore structures of **a**, **b**, TBAP-α and **c**, **d**, MeTBAP-α. The pore surfaces in **b** and **d** were created with a 1.2 A probe. The 1-D pores in TBAP-α are lined with polar carboxylic acid groups, **a**, while the 2-D pores in MeTBAP-α are not (Fig. 2g, main text).

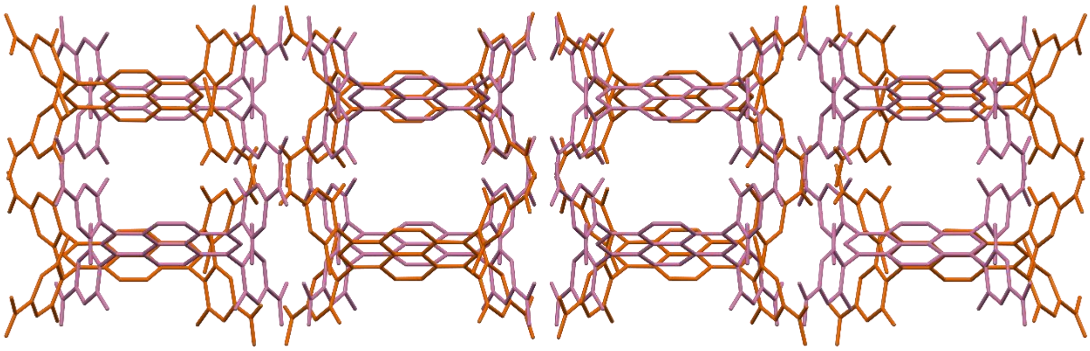

Figure S16. An overlay of the structures of MeTBAP-α (pink, single crystal structure) and diMeTBAP-α (orange, lowest-energy CSP-derived structure); both structures are essentially isomorphous and have the same characteristic 2-D pore layers, with the hydrogen-bonded carboxylic acids isolated between these layers.

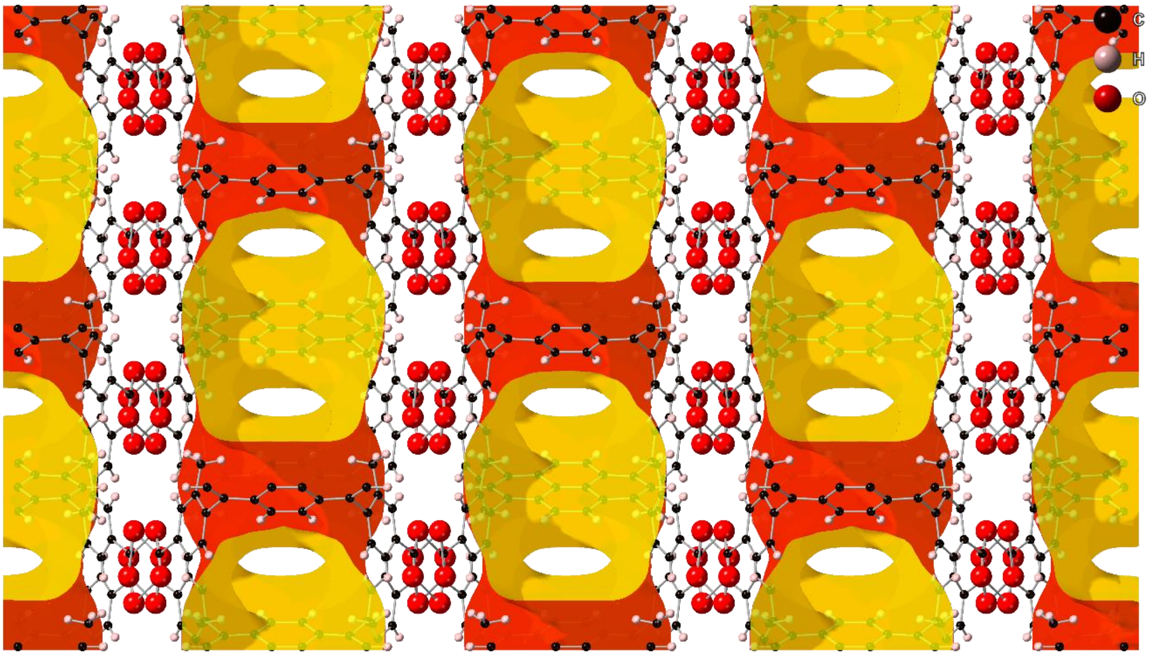

Figure S17. MeTBAP-α is linked by hydrogen-bonded carboxylic acid groups that sit outside the pore structure of the network.

S4.5 Thermogravimetric analysis

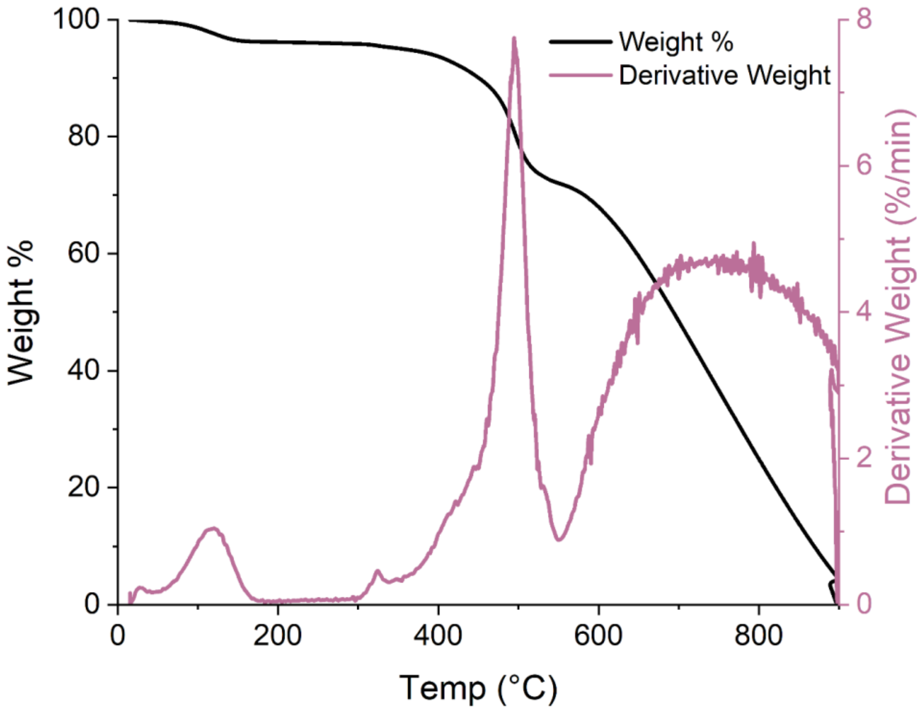

Figure S18. Thermogravimetric analysis of MeTBAP-α, demonstrating thermal stability up to 300 °C.

S5. Gas sorption data

S5.1 Nitrogen isotherm for MeTBAP-α after acid treatment (HCl)

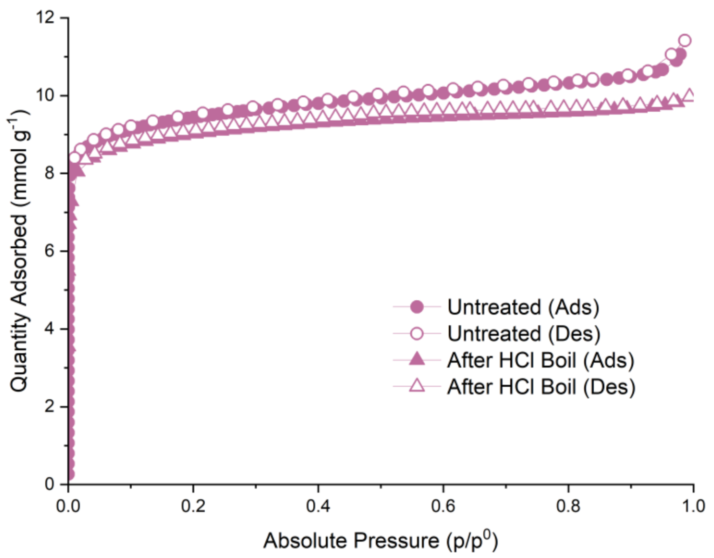

Figure S19. The nitrogen isotherms (77 K) for MeTBAP-α before and after exposure to boiling 1 M HCl overnight.

S5.2 Nitrogen isotherm for MeTBAP-α at 298 K

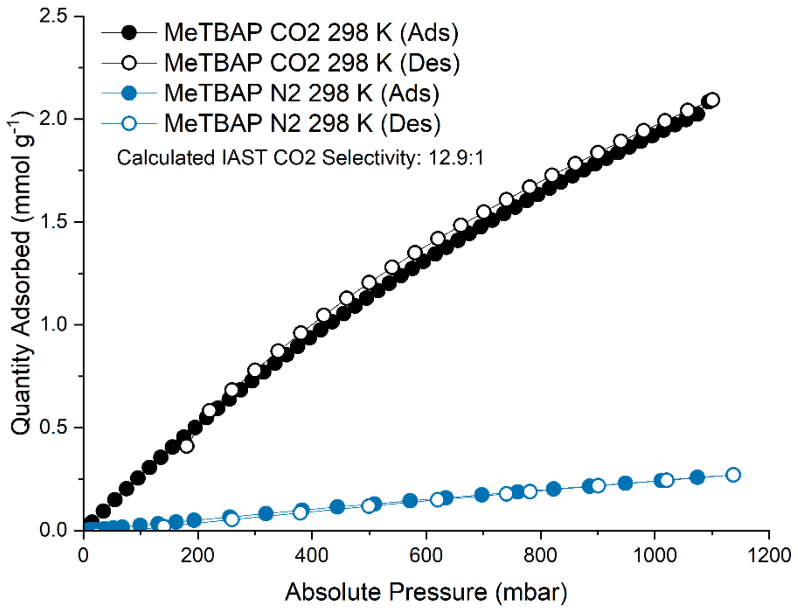

Figure S20. The nitrogen and CO_2 isotherms (298 K) for MeTBAP-α.

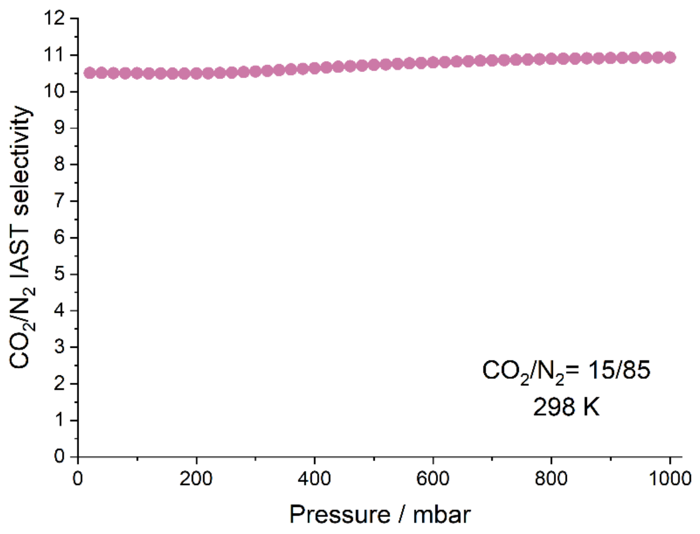

Figure S21. The IAST selectivity plot for CO_2 and N_2 isotherms (CO_2/N_2 = 15/85, 298 K) for MeTBAP-α. Calculated using pyGAPS.[35]

S5.3 CO_2 isotherms for HOFs at 195, 263, 283, 313 and 333 K

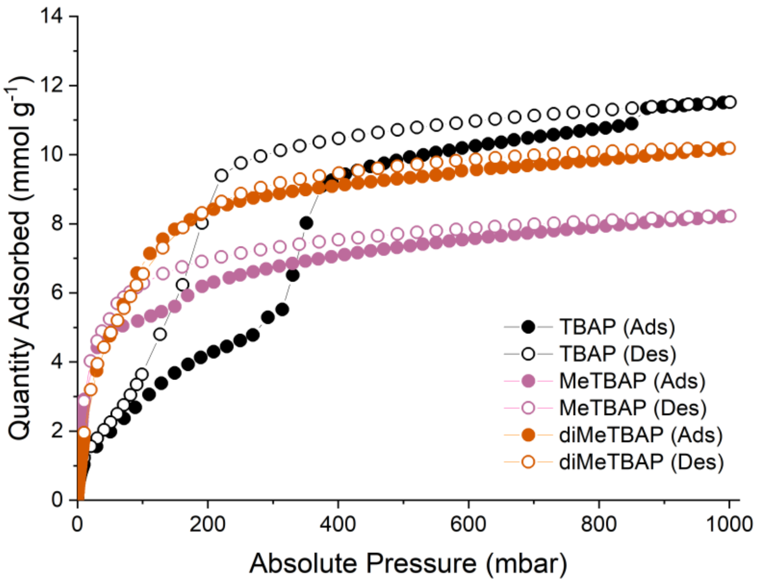

Figure S22. Dry CO_2 isotherms at 195 K for all HOFs.

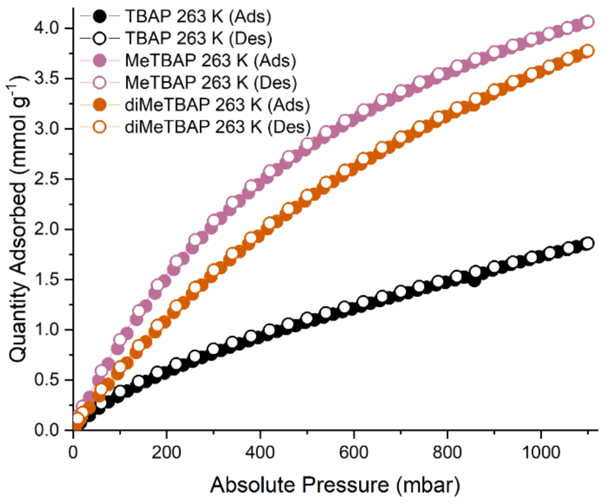

Figure S23. CO_2 isotherms at 263 K for all HOFs.

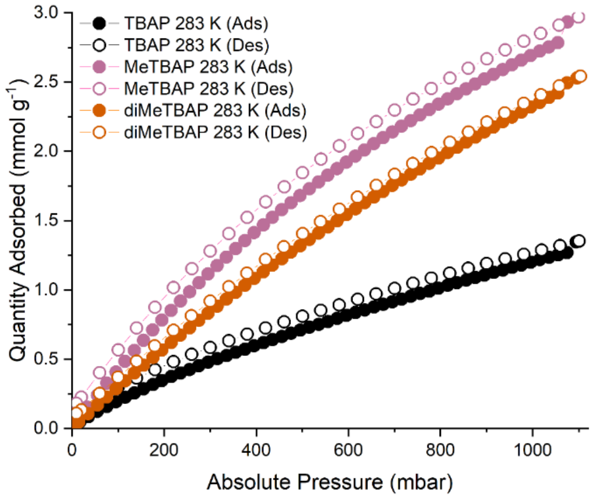

Figure S24. CO_2 isotherms at 283 K for all HOFs.

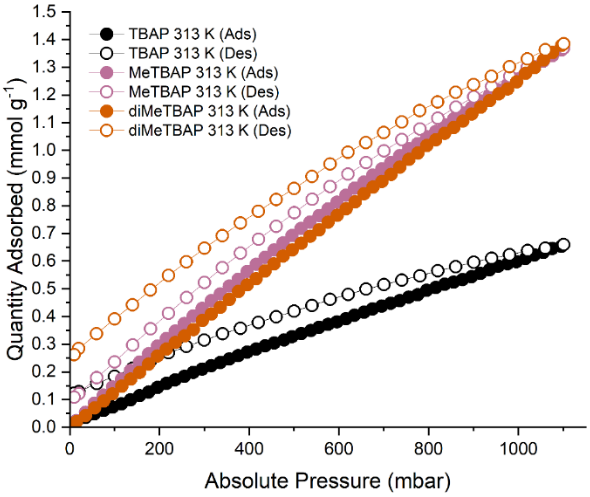

Figure S25. CO_2 isotherms at 313 K for all HOFs.

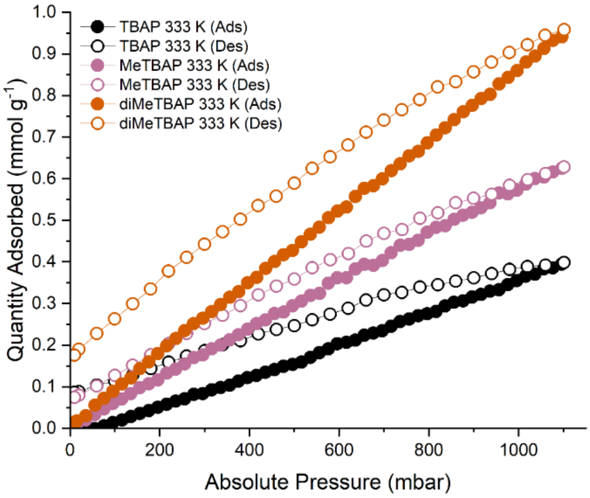

Figure S26. CO_2 isotherms at 333 K for all HOFs.

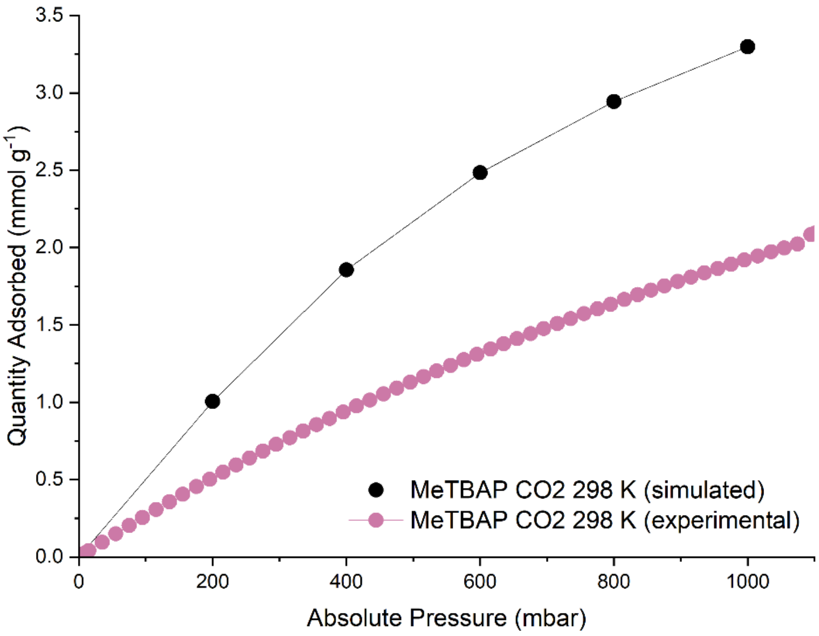

Figure 27. A comparison of the simulated and experimental CO_2 uptake of MeTBAP-α at 298 K.

S5.4 Heat of adsorption data

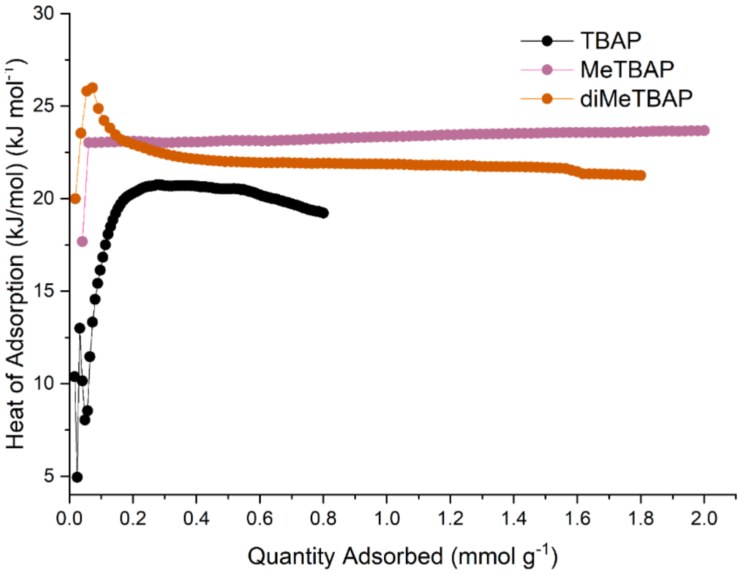

Figure S28. Heat of adsorption, as calculated from CO_2 isotherms from 0 to 1,000 mbar at 263, 273, 283, 298, 313 and 333 K for all HOFs.

S5.5 Pore Volumes

Table S3. A comparison of simulated and experimental data for the micropore volumes and SA_{BET} of TBAPy-α, MeTBAP-α and diMeTBAP-α.

Material	Micropore Volume/ $cm^3 g^{-1}$		$SA_{BET}/m^2 g^{-1}$	
	Simulated	Experimental	Simulated	Experimental

**TBAPy-α**	0.78	0.32	1731	1256
**MeTBAP-α**	0.24	0.26	1061	856
**diMeTBAP-α**	0.25	0.29	1106	1104

S6. Additional dynamic column breakthrough (DCB) data

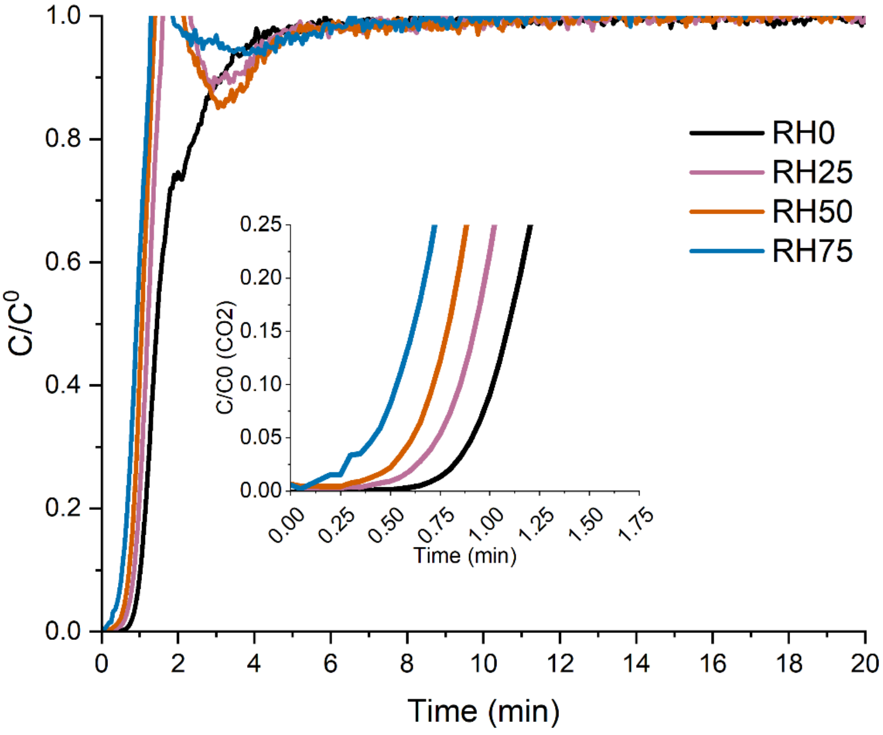

Figure S29. DCB traces for TBAP, illustrating the reduction in CO_2 breakthrough time in different relative humidities. Note that we were not able to get rid of the pressure spikes observed for this material.

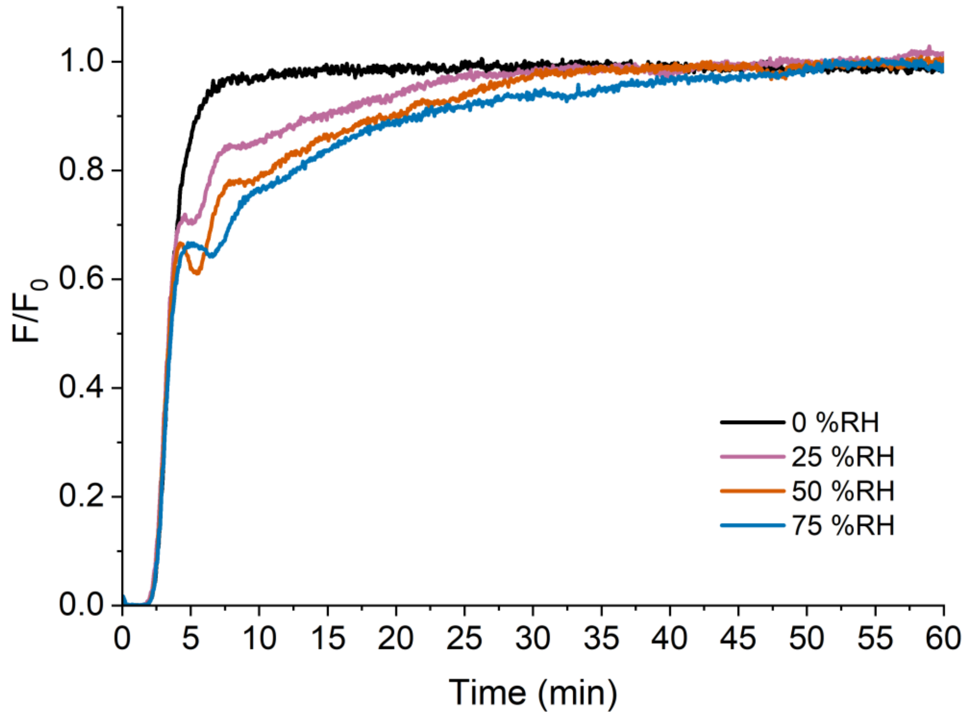

Figure S30. Breakthrough curve of 5% CO_2 in nitrogen at humidities in the range 0–75 %RH.

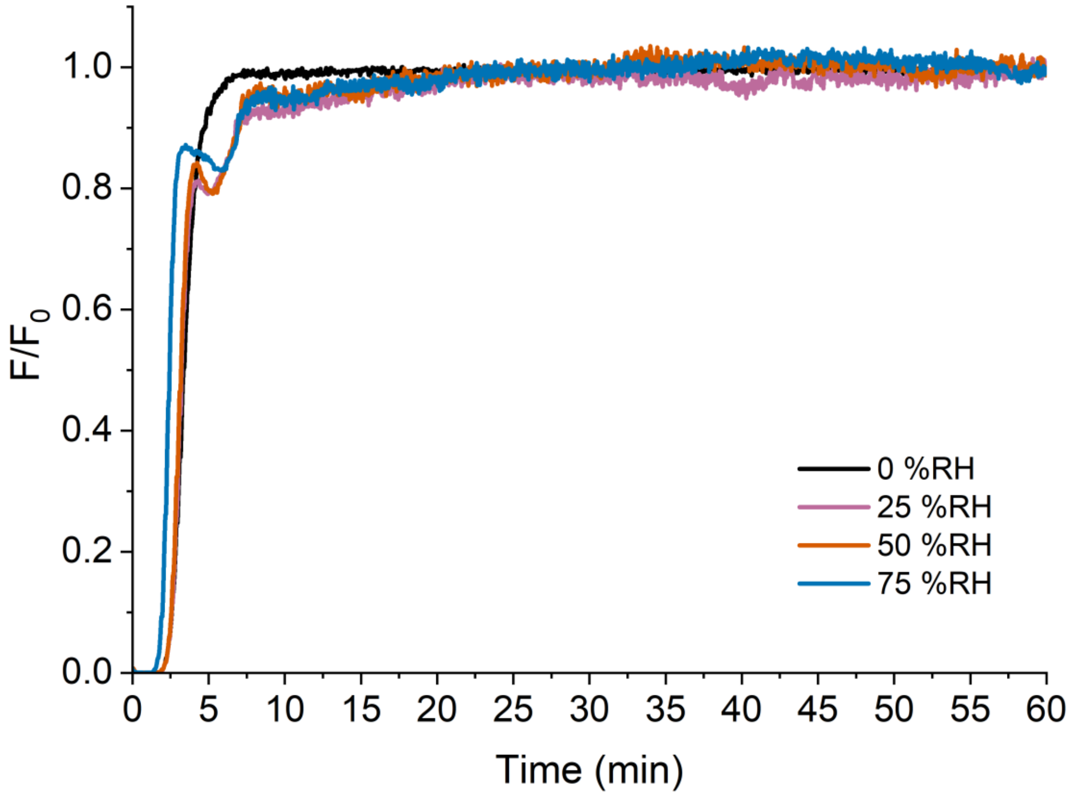

Figure S31: Breakthrough curve of 10% CO_2 in nitrogen at humidities in the range 0-75 %RH.

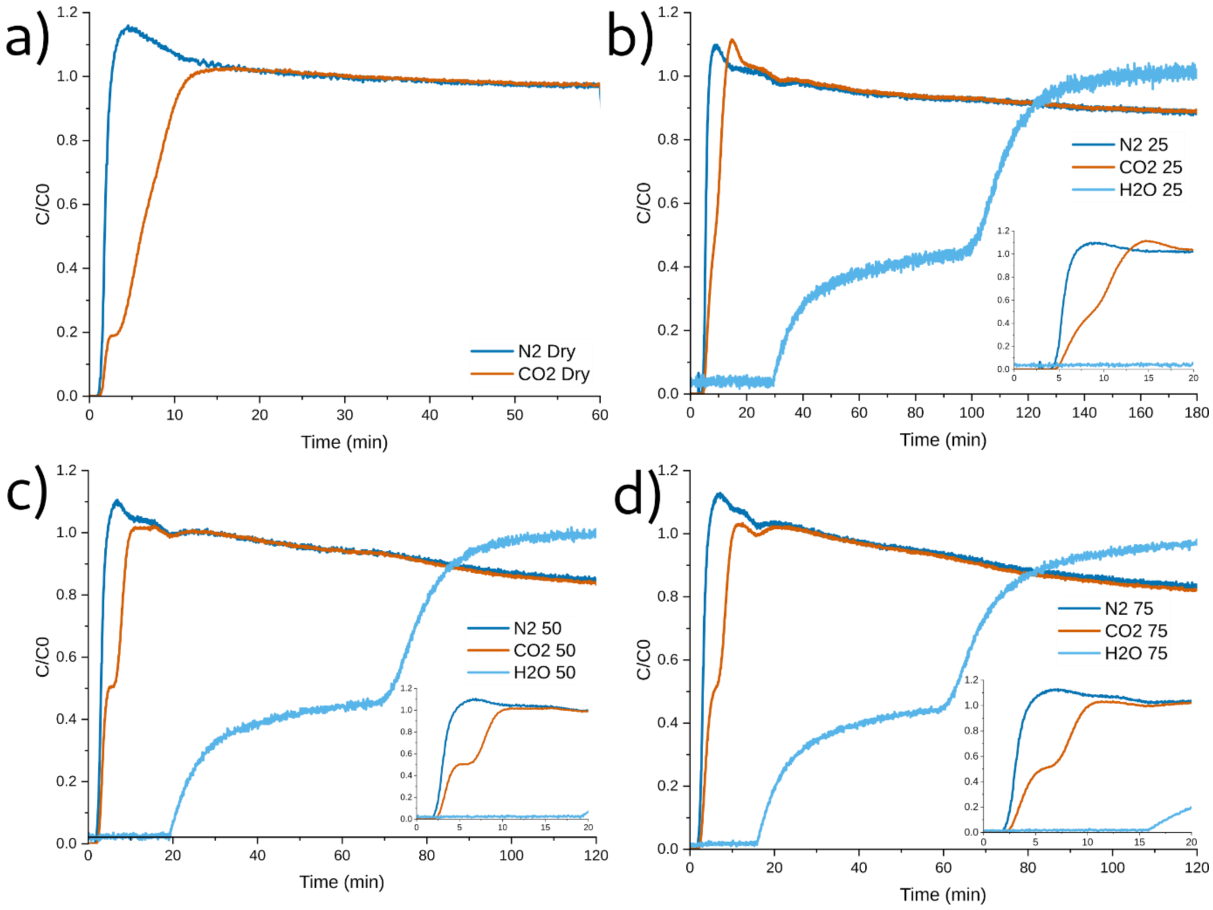

Figure S32. Competitive DCB traces of MeTBAP-α without presaturation.

Table S4. The calculated water and nitrogen capacities for MeTBAP-α from the above DCB traces.

Relative Humidity	Measured H2O Uptake from Isotherm/ mmol g^{-1}	Calculated H_2O Uptake from DCB/ mmol g^{-1}
**25**	0.23	0.21
**50**	0.34	0.31
**75**	0.60	0.58

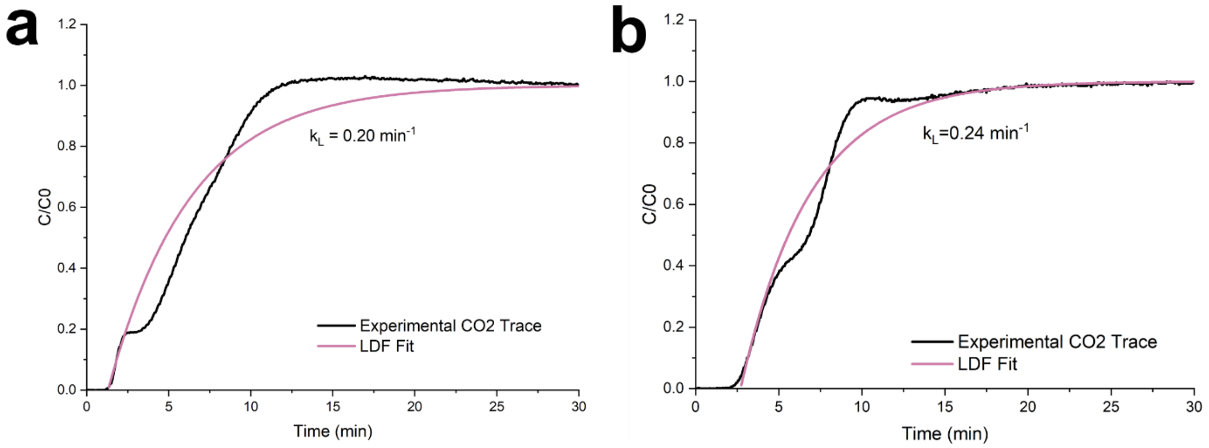

Figure S33. Linear driving force model fitting of breakthrough experiments at a) 0% and b) 75% RH.

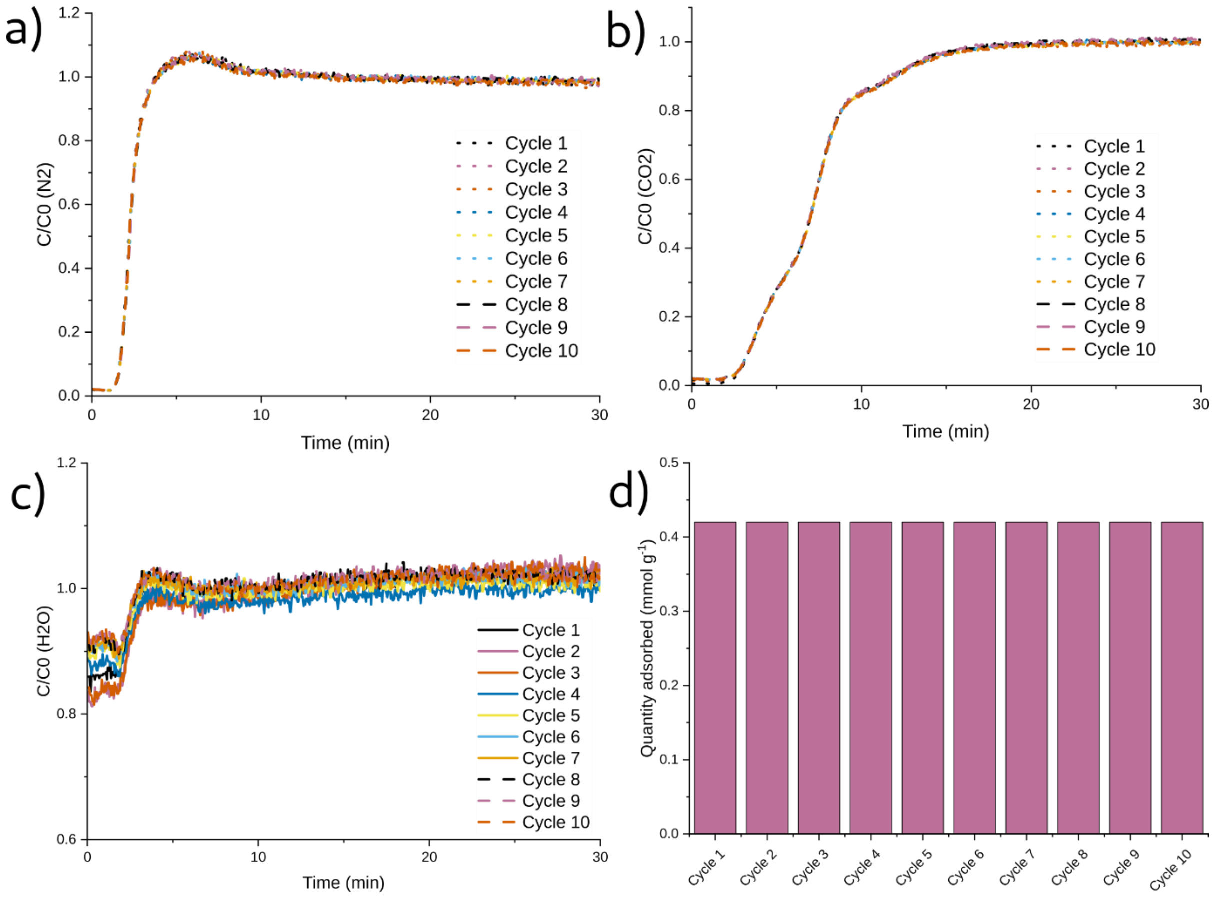

Figure S34. Breakthrough curves for a) N_2, b) CO_2 and c) H_2O over repeated cycles of 15% CO_2 in nitrogen at a humidity of 75 %RH as well as d) the calculated CO_2 uptakes for each cycle.

S7. Performance comparisons with other frameworks in the literature

S7.1 Comparison of CO_2-H_2O selectivity (separate isotherms)

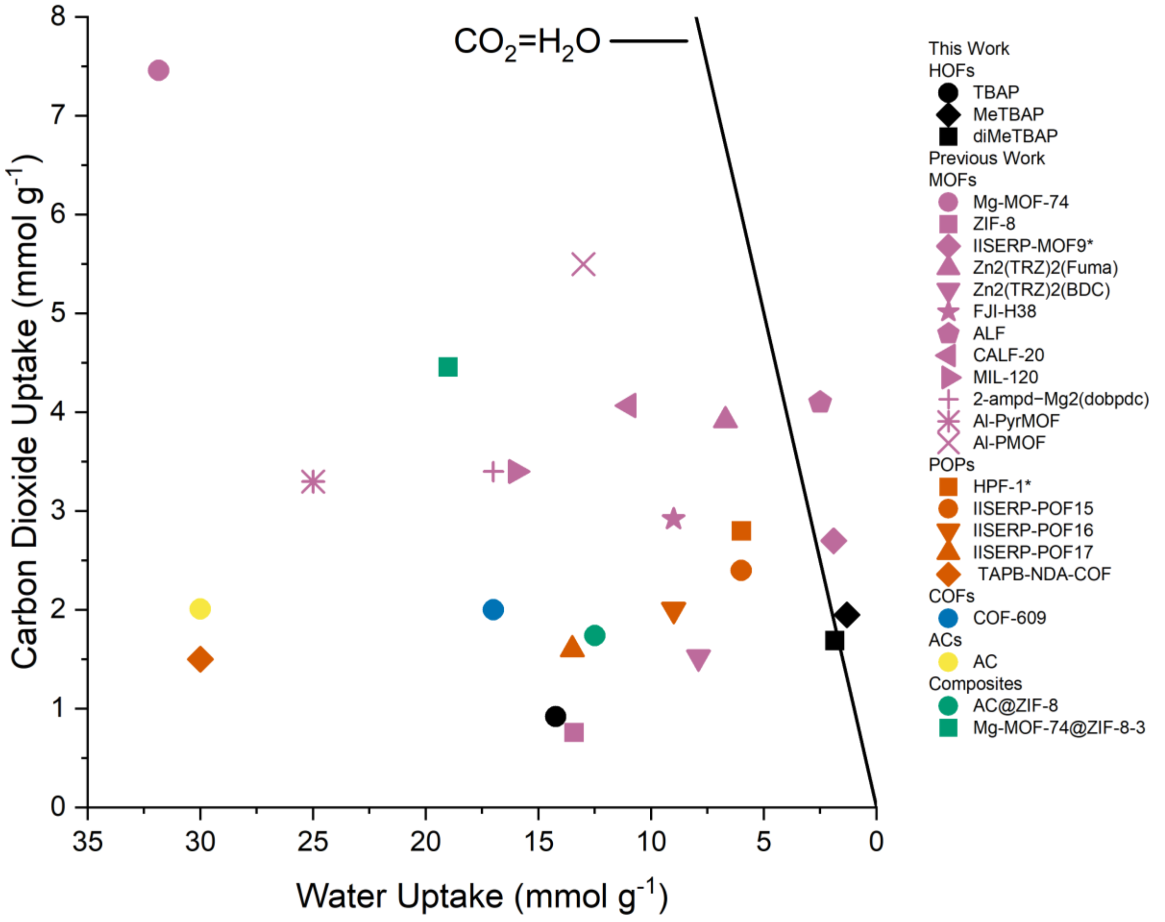

Figure S35. A comparison of materials with reported CO_2 (298 K, 1,000 mbar) and H_2O (298 K, 32 mbar) isotherms in the literature. Very few materials approach or cross CO_2=H_2O line, which represents materials where the CO_2 uptake is greater than or equal to the water uptake under these conditions. Of the three materials that do cross this line (MeTBAP-α, IISERP-MOF9, and ALF), the pyrene HOF has the lowest water uptake among these materials (Table S5).

Table S5. A comparison of materials with reported CO_2 (298 K, 1,000 mbar) and H_2O (298 K, 32 mbar) isotherms in the literature. MeTBAP-α has the lowest water uptake reported here.

Material	Carbon Dioxide Uptake/ mmol g^{-1}	Water Uptake/ mmol g^{-1}	CO_2/H_2O	Reference
**TBAP-α**	0.92	14.24	0.065	This Work
**MeTBAP-α**	1.95	1.30	0.662	This Work
**diMeTBAP-α**	1.69	1.85	0.914	This Work
**Mg-MOF-74**	7.46	31.85	0.234	[36]
**ZIF-8**	4.46	13.42	0.332	[36]
**IISERP-MOF9**	2.7	1.9	1.421	[37]
**Zn2(TRZ)2(Fuma)**	3.92	6.7	0.585	[38]
**Zn2(TRZ)2(BDC)**	1.52	7.9	0.192	[39]
**FJI-H38**	2.92	9	0.324	[40]
**ALF**	4.1	2.5	1.640	[40]
**CALF-20**	4.07	11	0.370	[41]
**MIL-120**	3.4	16	0.213	[42]
**2-ampd−Mg2(dobpdc)**	3.4	17	0.200	[43]
**Al-PyrMOF**	3.3	25	0.132	[44]
**Al-PMOF**	5.5	13	0.423	[44]
**HPF-1***	2.8	6	0.467	[45]
**IISERP-POF15**	2.4	6	0.400	[46]
**IISERP-POF16**	2	9	0.222	[46]
**IISERP-POF17**	1.6	13.5	0.119	[46]
**TAPB-NDA-COF**	1.5	30	0.050	[25]
**COF-609**	2	17	0.118	[47]
**AC**	2.01	30	0.067	[48]
**AC@ZIF-8**	1.74	12.5	0.139	[48]
**Mg-MOF-74@ZIF-8-3**	0.76	19.01	0.040	[34]

S7.2 Comparison of wet versus dry CO_2 uptake in breakthrough experiments

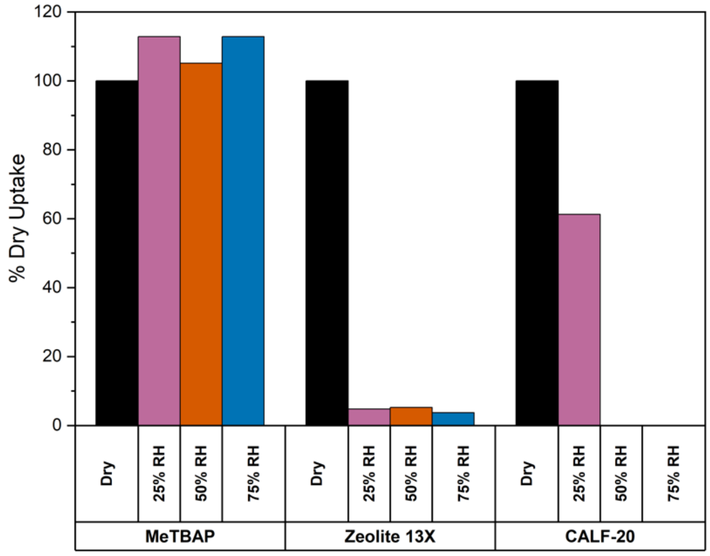

Figure S36. Comparison of the effects of humidity on the CO_2 uptakes relative to dry uptake of MeTBAP-α, Zeolite 13X and CALF-20 at a CO_2 partial pressure of 150 mbar and a temperature of 298 K. MeTBAP-α and Zeolite-13X data were calculated from DCB experiments performed as part of this work. CALF-20 data is from recent work by Moreton *et al.*[48]

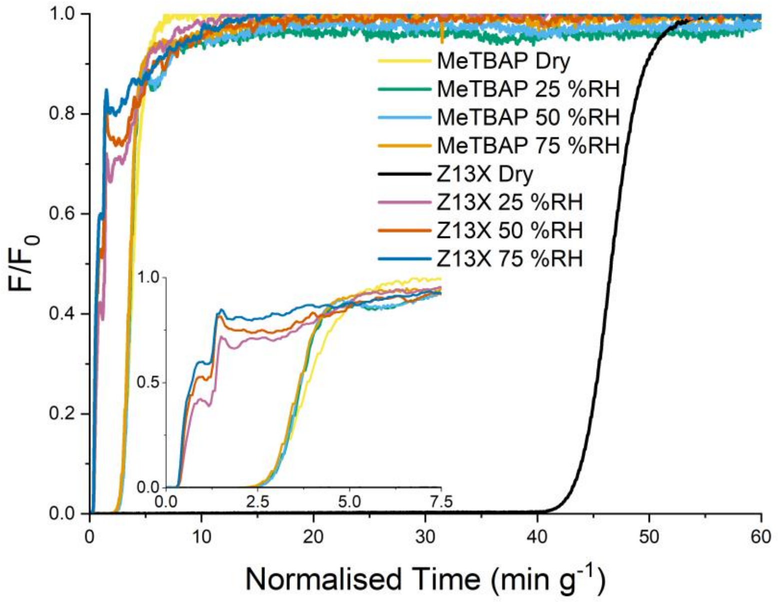

Figure S37. The raw breakthrough data of Z13X and MeTBAP-α at a CO_2 partial pressure of 150 mbar and a temperature of 298 K.

S7.3 Comparison of Binding Energies

Table S6: A comparison of the calculated CO_2 and H_2O binding energies of MeTBAP-α and diMeTBAP-α and some famous examples from the literature.

	E_{CO2}	$E_{CO2-H2O}$	Ref

**diMeTBAP-α MeTBAP-α**	-0.223	0.107	This work
**Mg-MOF-74**	-0.157	0.139	[34]
**IISERP-MOF9**	-0.224	0.313	[36]
**ALF**	-0.236	0.255	[40]
**CALF-20**	-0.285/-0.209	0.263/0.236	[41]
**Al-PyrMOF**	-0.340	0.308	[44]
**COF-609**	-0.438	0.406	[47]

S8. References

1 https://www.elsevier.com/solutions/reaxys.

2 Liu, T.; Hang, Q.; Harding, S. D.; Borne, I.; Chen, L.; Ward, J. W.; Weston, S. C.; Cooper, A. I. Bottom-Up Computational Design of Shape-Selective Organic Macrocycles for Humid CO2 Capture. 2024, preprint, *ChemRxiv,* 1-21, DOI: 10.26434/chemrxiv-2024-5vwn0.

3 C. Bannwarth, S. Ehlert and S. Grimme, *J. Chem. Theory Comput.*, 2019, **15**, 1652–1671.

4 S. Grimme, C. Bannwarth and P. Shushkov, *J. Chem. Theory Comput.*, 2017, **13**, 1989–2009.

5 J.-L. Calais, *Int. J. Quantum Chem.*, 1993, **47**, 101–101.

6 R. G. Parr, in *Horizons of Quantum Chemistry*, eds. K. Fukui and B. Pullman, Springer Netherlands, Dordrecht, 1980, pp. 5–15.

7 A. D. Becke, *J. Chem. Phys.*, 1997, **107**, 8554–8560.

8 H. L. Schmider and A. D. Becke, *J. Chem. Phys.*, 1998, **108**, 9624–9631.

9 F. Weigend and R. Ahlrichs, *Phys. Chem. Chem. Phys.*, 2005, **7**, 3297–3305.

10 S. Grimme, S. Ehrlich and L. Goerigk, *J. Comput. Chem.*, 2011, **32**, 1456–1465.

11 M. J. Frisch, G. W. Trucks, H. B. Schlegel, G. E. Scuseria, M. A. Robb, J. R. Cheeseman, G. Scalmani, V. Barone, G. A. Petersson, H. Nakatsuji, X. Li, M. Caricato, A. V. Marenich, J. Bloino, B. G. Janesko, R. Gomperts, B. Mennucci, H. P. Hratchian, J. V. Ortiz, A. F. Izmaylov, J. L. Sonnenberg, Williams, F. Ding, F. Lipparini, F. Egidi, J. Goings, B. Peng, A. Petrone, T. Henderson, D. Ranasinghe, V. G. Zakrzewski, J. Gao, N. Rega, G. Zheng, W. Liang, M. Hada, M. Ehara, K. Toyota, R. Fukuda, J. Hasegawa, M. Ishida, T. Nakajima, Y. Honda, O. Kitao, H. Nakai, T. Vreven, K. Throssell, J. A. Montgomery Jr., J. E. Peralta, F. Ogliaro, M. J. Bearpark, J. J. Heyd, E. N. Brothers, K. N. Kudin, V. N. Staroverov, T. A. Keith, R. Kobayashi, J. Normand, K. Raghavachari, A. P. Rendell, J. C. Burant, S. S. Iyengar, J. Tomasi, M. Cossi, J. M. Millam, M.

Klene, C. Adamo, R. Cammi, J. W. Ochterski, R. L. Martin, K. Morokuma, O. Farkas, J. B. Foresman and D. J. Fox, Gaussian 16 Rev. A.03 2016.

12 B. Delley, *J. Chem. Phys.*, 2000, **113**, 7756–7764.

13 Materials Studio DS BIOVIA, San Diego 2017.

14 J. P. Perdew, K. Burke and M. Ernzerhof, *Phys. Rev. Lett.*, 1996, **77**, 3865–3868.

15 S. L. Mayo, B. D. Olafson and W. A. Goddard, *J. Phys. Chem.*, 1990, **94**, 8897–8909.

16 D. Frenkel and B. Smit, *Understanding Molecular Simulation: from algorithms to applications.*, Academic Press, San Diego, 2002.

17 D. Dubbeldam, S. Calero, D. E. Ellis and R. Q. Snurr, *Mol. Simul.*, 2016, **42**, 81–101.

18 T. A. Manz and N. Gabaldon Limas, Chargemol program for performing DDEC analysis (version 3.5) ddec.sourceforge.net 2017.

19 T. A. Manz and N. G. Limas, *RSC Adv.*, 2016, **6**, 47771–47801.

20 N. G. Limas and T. A. Manz, *RSC Adv.*, 2016, **6**, 45727–45747.

21 T. D. Kühne, M. Iannuzzi, M. Del Ben, V. V. Rybkin, P. Seewald, F. Stein, T. Laino, R. Z. Khaliullin, O. Schütt, F. Schiffmann, D. Golze, J. Wilhelm, S. Chulkov, M. H. Bani-Hashemian, V. Weber, U. Borštnik, M. Taillefumier, A. S. Jakobovits, A. Lazzaro, H. Pabst, T. Müller, R. Schade, M. Guidon, S. Andermatt, N. Holmberg, G. K. Schenter, A. Hehn, A. Bussy, F. Belleflamme, G. Tabacchi, A. Glöß, M. Lass, I. Bethune, C. J. Mundy, C. Plessl, M. Watkins, J. VandeVondele, M. Krack and J. Hutter, *J. Chem. Phys.*, 2020, **152**, 194103.

22 J. J. Potoff and J. I. Siepmann, *AIChE Journal*, 2001, **47**, 1676–1682.

23 C. Plett and S. Grimme, *Angew. Chem. Int. Ed.*, 2023, **62**, e202214477.

24 C. Bannwarth, E. Caldeweyher, S. Ehlert, A. Hansen, P. Pracht, J. Seibert, S. Spicher and S. Grimme, *WIREs Comput. Mol. Sci.*, 2021, **11**, e1493.

25 H. Veldhuizen, S. A. Butt, A. van Leuken, B. van der Linden, W. Rook, S. van der Zwaag and M. A. van der Veen, *ACS Appl. Mater. Interfaces*, 2023, **15**, 29186–29194.

26 G. M. Sheldrick, *Acta Crystallogr. A*, 2015, **71**, 3–8.

27 G. M. Sheldrick, *Acta Crystallogr. C*, 2015, **71**, 3–8.

28 O. V. Dolomanov, L. J. Bourhis, R. J. Gildea, J. A. K. Howard and H. Puschmann, *J. Appl. Crystallogr.*, 2009, **42**, 339–341.

29 A. L. Spek, *Acta Crystallogr. C*, 2015, **71**, 9–18.

30 A. L. Spek, *J. Appl. Crystallogr.*, 2003, **36**, 7–13.

31 J. Rouquerol, F. Rouquerol, C. Pérès, Y. Grillet and M. Boudellal, in *Characterisation of Porous Solids: Proceedings of a Symposium Held at the Université de Neuchâtel, Switzerland, from 9 to 12 July 1978*, eds. S. J. Gregg, K. S. W. Sing and H. F. Stoeckli, Society of Chemical Industry, London, 1979, p. 107.

32 B.H. Toby & R.B. Von Dreele, J. Appl. Cryst., 2013, 46, 544-549

33 N. S. Wilkins, A. Rajendran and S. Farooq, *Adsorption*, 2021, **27**, 397–422.

34 Sircar, S.; Hufton, J. R. Why Does the Linear Driving Force Model for Adsorption Kinetics Work? Adsorption, 2000, 6, 137–147. DOI: 10.1023/A:1008965317983

35 P. Iacomi, P. L. Llewellyn, *Adsorption,* 2019, **25**, 1533–1542

36Y. Wan, D. Kong, F. Xiong, T. Qiu, S. Gao, Q. Zhang, Y. Miao, M. Qin, S. Wu, Y. Wang, R. Zhong and R. Zou, *Chin. J. Chem. Eng.*, 2023, **61**, 82-89

37 S. Nandi, S. Haldar, D. Chakraborty and R. Vaidhyanathan, *J. Mater. Chem. A*, 2017, **5**, 535–543.

38 Y. M. Gu, Y. H. Wang, S. S. Zhao, H. J. Fan, X. W. Liu, Z. Lai and S. D. Wang, *Fuel*, 2023, **336**, 126793.

39 D. Song, F. Jiang, D. Yuan, Q. Chen and M. Hong, *Small*, 2023, **2302677**, 1–10.

40 H. A. Evans, D. Mullangi, Z. Deng, Y. Wang, S. B. Peh, F. Wei, J. Wang, C. M. Brown, D. Zhao, P. Canepa and A. K. Cheetham, *Sci. Adv.*, 2022, **8**, 1473–1484.

41 J. Bin Lin, T. T. T. Nguyen, R. Vaidhyanathan, J. Burner, J. M. Taylor, H. Durekova, F. Akhtar, R. K. Mah, O. Ghaffari-Nik, S. Marx, N. Fylstra, S. S. Iremonger, K. W. Dawson, P. Sarkar, P. Hovington, A. Rajendran, T. K. Woo and G. K. H. Shimizu, *Science*, 2021, **374**, 1464–1469.

42 R. P. Loughran, T. Hurley, A. Gładysiak, A. Chidambaram, K. Khivantsev, E. D. Walter, T. R. Graham, P. Reardon, J. Szanyi, D. B. Fast, Q. R. S. Miller, A.-H. A. Park and K. C. Stylianou, *Cell Rep. Phys. Sci.*, 2023, **4**, 101470.

43 R. L. Siegelman, P. J. Milner, A. C. Forse, J.-H. Lee, K. A. Colwell, J. B. Neaton, J. A. Reimer, S. C. Weston and J. R. Long, *J. Am. Chem. Soc.*, 2019, **141**, 13171–13186.

44 P. G. Boyd, A. Chidambaram, E. García-Díez, C. P. Ireland, T. D. Daff, R. Bounds, A. Gładysiak, P. Schouwink, S. M. Moosavi, M. M. Maroto-Valer, J. A. Reimer, J. A. R. Navarro, T. K. Woo, S. Garcia, K. C. Stylianou and B. Smit, *Nat.*, 2019, **576**, 253–256.

45 S. Nandi, U. Werner-Zwanziger and R. Vaidhyanathan, *J. Mater. Chem. A*, 2015, **3**, 21116–21122.

46 H. D. Singh, P. Singh, D. Rase and R. Vaidhyanathan, *Mater. Adv.*, 2023, **4**, 3055–3060.

47 H. Lyu, H. Li, N. Hanikel, K. Wang and O. M. Yaghi, *J. Am. Chem. Soc.*, 2022, **144**, 12989–12995.

48 Y. Ji, X. Liu, H. Li, X. Jiao, X. Yu and Y. Zhang, *J. Ind. Eng. Chem.*, 2023, **121**, 331–337.

48 J. C. Moreton, R. Krishna, J. van Baten, N. Fylstra, M. Chen, T. Carr, K. Fielder, K. Chan, G. K. H. Shimizu and S. Yamamoto, *ChemRxiv*, 2024, preprint, DOI: doi:10.26434/chemrxiv-2024-0mhg1.
